# Ultra‐Tough Elastomers from Stereochemistry‐Directed Hydrogen Bonding in Isosorbide‐Based Polymers

**DOI:** 10.1002/anie.202115904

**Published:** 2022-03-04

**Authors:** Shannon R. Petersen, Hannah Prydderch, Joshua C. Worch, Connor J. Stubbs, Zilu Wang, Jiayi Yu, Maria C. Arno, Andrey V. Dobrynin, Matthew L. Becker, Andrew P. Dove

**Affiliations:** ^1^ Department of Polymer Science The University of Akron Akron OH 44224 USA; ^2^ School of Chemistry University of Birmingham Birmingham B15 2TT UK; ^3^ Department of Chemistry University of North Carolina Chapel Hill Chapel Hill NC, 27599 USA; ^4^ Department of Chemistry, Mechanical Engineering and Materials Science Biomedical Engineering and Orthopedic Surgery Duke University Durham NC, 20899 USA

**Keywords:** Elastomers, Isomannide, Isosorbide, Polyurethane, Stereochemistry

## Abstract

The remarkable elasticity and tensile strength found in natural elastomers are challenging to mimic. Synthetic elastomers typically feature covalently cross‐linked networks (rubbers), but this hinders their reprocessability. Physical cross‐linking via hydrogen bonding or ordered crystallite domains can afford reprocessable elastomers, but often at the cost of performance. Herein, we report the synthesis of ultra‐tough, reprocessable elastomers based on linear alternating polymers. The incorporation of a rigid isohexide adjacent to urethane moieties affords elastomers with exceptional strain hardening, strain rate dependent behavior, and high optical clarity. Distinct differences were observed between isomannide and isosorbide‐based elastomers where the latter displays superior tensile strength and strain recovery. These phenomena are attributed to the regiochemical irregularities in the polymers arising from their distinct stereochemistry and respective inter‐chain hydrogen bonding.

## Graphical abstract

The stereochemical configurations of the rigid‐ring structures of isosorbide (ISPU) and isomannide (IMPU) direct strong hydrogen‐bonding interactions which impart unique properties including strain‐rate‐dependent behavior, significant strain hardening, and high optical clarity that are not possible using other chemical approaches to elastomer design.

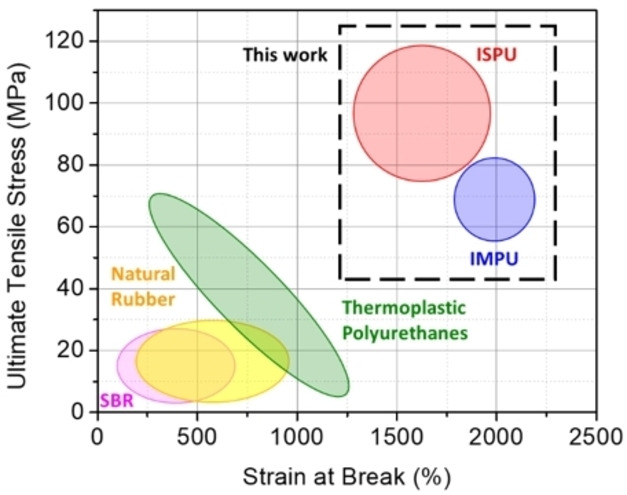

## Introduction

Elastomers have become ubiquitous throughout society since Charles Goodyear first described the vulcanization of natural rubber in 1844.^[1]^ Owing to their high fracture strength, toughness, and extensibility, elastomers have found widespread utility in the automotive industry, healthcare, coatings, and robotics.^[2]^ While there remains an ever increasing demand for tough, high modulus materials, the long term impacts of modern elastomers on the environment are a significant concern.[^2c^, ^3^] Traditional strategies to increase mechanical strength involve chemical crosslinking to form rubbers or thermosets with a concomitant increase in the crosslink density of the material providing increased strength.[^2c^, ^3a^, ^3c^, ^4^] While covalent chemical crosslinks impart good solvent resistance, high thermal stability and toughness, they severely limit end‐of‐life options.^[3b]^ Furthermore, most commercially relevant rubbers are derived from non‐renewable, petrochemical feedstocks, further detracting from sustainability.^[2d]^ While rubbers derived from renewable sources have yielded examples of degradable, crosslinked materials,^[5]^ their overall performance generally lags behind non‐renewable counterparts.

Thermoplastic elastomers (TPEs), by contrast, derive their elasticity from physically‐crosslinked networks, including crystalline domains and hydrogen bonding, which allow these materials to be reprocessed at elevated temperatures, reducing their environmental impact.[^2d^, ^6^] Conventional TPEs utilize interplay between hard blocks and soft blocks to access diverse material properties.^[7]^ Therefore, strength and elasticity exist in a competitive tradeoff, i.e. increasing the hard block content improves material strength but reduces extensibility,[^2b^, ^3a^] making it challenging to access strong and highly extensible materials. As with rubbers, the majority of modern TPEs are also derived from non‐renewables. Attempts to increase the sustainability of TPEs have largely focused on incorporation of renewable chain‐extenders^[8]^ and diisocyanate precursors into thermoplastic polyurethanes. Recent strategies to access more sustainable TPEs have turned to non‐polyurethane‐based block polymers such as tri‐block polymers formed of polyesters,^[9]^ polycarbonates^[10]^ or polyacrylics^[11]^ which are derived from renewable feedstocks.

As one of the top 20 biomass‐sourced molecules, isosorbide has been the focus of much attention in polymer synthesis,^[12]^ and provides a renewable feedstock alternative to petroleum derivatives for commercial polymer production that is available at scale.[^8a^, ^12b^, ^12c^, ^12e^] The majority of polymers that contain isosorbide units have been shown to be rigid thermoplastics with high glass transition temperatures (*T*_g_), excellent durability and high optical clarity.[^12a^, ^12b^, ^12c^] Indeed, Mitsubishi has produced an isosorbide‐based polycarbonate, DURABIO^TM^, which is used in the front panels of smartphones.[^12c^, ^13^] Linear isosorbide‐containing polyethers^[14]^ and polyesters^[15]^ without block‐like architectures have displayed elastomeric profiles, but only in statistical copolymer samples. In these cases, the elasticity was hypothesized to arise from transient hydrogen‐bonding^[15c]^ networks. Moreover, these examples display comparable properties to conventional multi‐block polyurethane structures where isosorbide was used as the chain‐extender.[^8b^, ^8d^] Other reports have shown that the dynamic rupture and reformation of the reversible bonds releases residual stress and promotes effective energy dissipation, simultaneously enhancing both the strength and elasticity of the material.[^3a^, ^16^] A recent study reported thermoset isohexide polyesters, however the mechanical performance was typical for crosslinked materials and moreover, was inferior to a dilactone analogue.^[17]^

We have recently shown that regioregular isoidide (*exo*/*exo* isomer) and isomannide (*endo*/*endo* isomer) could be incorporated into alternating polyurethanes to create materials in which the stereochemistry of the isohexide determined if the material displayed plastic or elastic behavior.^[18]^ We were interested to study the effect of isosorbide on the resultant materials’ properties on account of its *endo*/*exo* stereochemistry that we postulated would break up the regioregularity of the other isohexide materials to potentially deliver materials with improved strength and/or ductility to either of the other isohexide isomer‐based polymers. Herein, we report that by creating isosorbide‐based polymers with high molar masses in alternating sequences, instead of the block structures found in traditional polyurethane TPEs, to thermoplastic elastomers with exceptional mechanical strength, extensibility and high optical clarity can be obtained. These outstanding properties are correlated to a supramolecular hydrogen‐bonding mechanism underpinning the strain‐hardening behavior. Contrasting the elastomeric behavior of the isosorbide polymer with an isomeric analogue formed from isomannide, that we have previously described,^[18]^ we demonstrate that the backbone regioirregularity that results from the *endo*/*exo* stereochemistry of isosorbide^[19]^ provides distinct advantages with respect to network dynamics that manifest in the elastic recovery behavior and superior toughness. Moreover, we demonstrate that this behavior results from the combination of the structurally rigid isosorbide unit being adjacent to a urethane group.

## Results and Discussion

**Figure 1:**
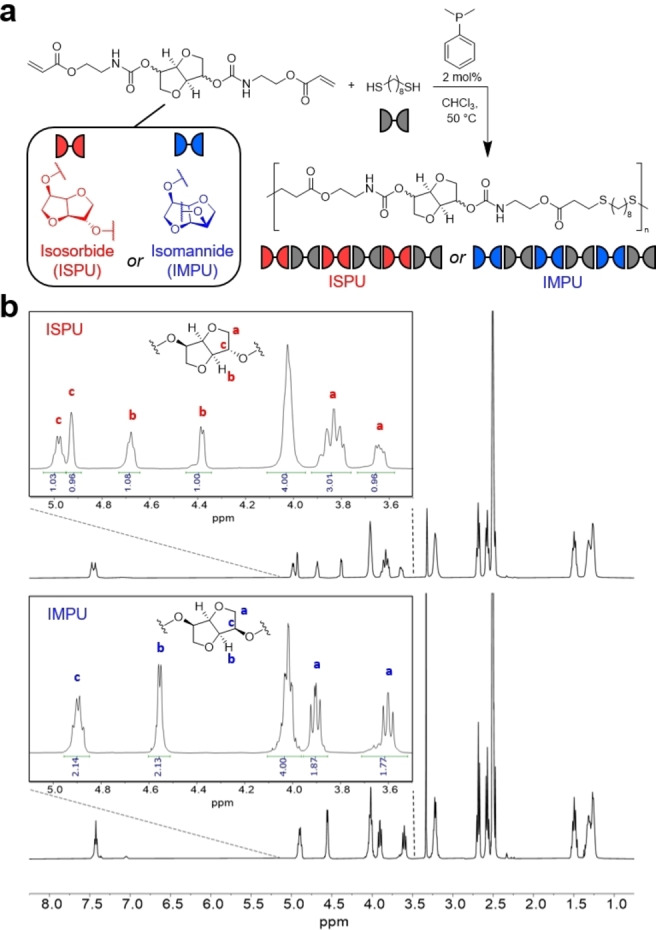
a) Thiol‐ene polymerization of acrylated isohexide isomers with defined stereochemistry and 1,8‐octanedithiol yielding isosorbide polyurethane (ISPU) or isomannide polyurethane (IMPU). b) ^1^H NMR spectra of ISPU and IMPU with inset figures highlighting the stereochemical differences.

Polyurethanes containing either isosorbide or isomannide moieties in an alternating sequence were synthesized via a phosphine‐mediated thiol‐ene addition polymerization in a comparable manner to that previously reported (Figure 1a).^[18]^ This efficient synthesis yields isosorbide polyurethane (ISPU) or isomannide polyurethane (IMPU) linear polymers with high molecular weight (*M*_w_=109.5 kDa and *M*_w_=56.5 kDa respectively) and alternating sequences as indicated by ^1^H NMR spectroscopic analysis (Figure 1b). The isolated polymers were readily processable by compression molding into transparent, thin films at 120 °C and were thermally stable (*T*_d_ onset >300 °C) despite the inclusion of a renewable sugar backbone in the materials. Differential scanning calorimetry (DSC) analysis of both ISPU and IMPU showed that they were completely amorphous, with glass transition temperatures (*T*_g_) below 25 °C. Thus, ISPU was anticipated to be flexible at room temperature and above. Employing dithiol comonomers with shorter aliphatic spacers also led to amorphous polymers, but the *T*_g_s approached ambient temperature resulting in variable tensile behavior with the emergence of yield points (Figure S29). It should be noted that all materials exhibit a single *T*_g_, which indicates that the materials are not phase separated, which could be beneficial to chain mobility.^[20]^

ISPU and IMPU were uniaxially strained at a strain rate of 10 mm min^−1^ to initially assess their mechanical properties. This revealed that the ISPU behaved comparably to IMPU,^[18]^ both as tough elastomers with high tensile strength and extensibility at failure. Although the molecular weight of ISPU was significantly higher than IMPU (*M*_w_=109.5 kDa and *M*_w_=56.5 kDa, respectively), a previous study showed consistent tensile behavior for IMPU irrespective of molecular weight (*M*_w_=95 kDa vs 40 kDa).^[18]^ This indicates that mechanical performance is not dependent on molecular weight, in the studied polymer length range, for these isohexide elastomers.

**Figure 2:**
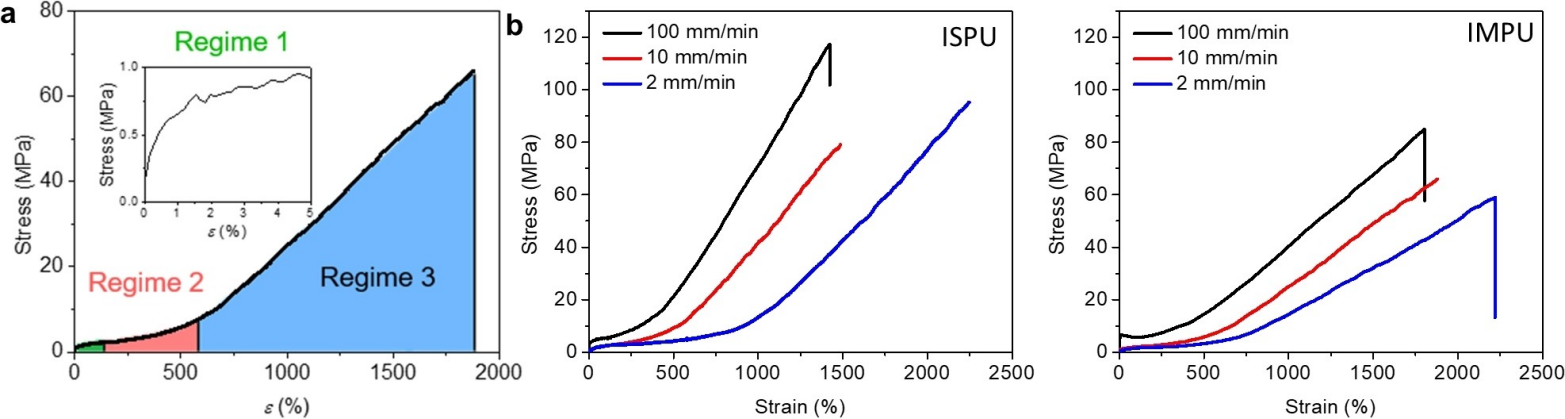
a) Representative stress vs. strain curve used to highlight the different strain dependent deformation regimes observed in ISPU and IMPU; b) Representative ISPU and IMPU tensile data comparing the effects of isohexide stereochemistry and deformation speed on mechanical performance (22 °C, *n*=3, annealed at 25 °C for 7 d after pressing).

ISPU was slightly stronger, with an average stress at break of 75.1 MPa, compared to 63.5 MPa in IMPU (Figure 2, Table 1). Conversely, IMPU had a higher strain at break than ISPU, with the materials breaking at 1806 % and 1466 % strain, respectively. Interestingly, both materials exhibited unique, strain‐dependent mechanical responses to deformation that resulted in “J‐shaped” stress‐strain curves with three distinct elastic regimes (Figure 2a). The first regime is characterized by a rapid, linear increase of stress at low strains (ϵ<0.05). In the second regime (approximately 0.05<*ϵ*<5.0), the stress continues to increase with increasing strain, but the rate of increase is significantly less compared to the other regimes. Pronounced strain hardening occurs in the third regime (*ϵ*>5.0) as the rate of stress accumulation increases again. Rheological analysis confirmed that both materials were well above the molecular weight necessary for chain entanglement (the complete details are included in Supporting Information). Hence, the variations in mechanical performance can be attributed to stereochemistry differences^[12e]^ between the isohexide units of ISPU and IMPU. However, the unique strain dependence observed in both materials suggests that they undergo similar network dynamics during deformation.

**Table 1:** Mechanical characterization of isosorbide and isomannide polyurethanes.

Polymer	Strain Rate [mm min^−1^]	Strain at Break [%]^[a]^	Stress at Break [MPa]^[a]^	*E*_1_ [MPa]^[b]^	*E*_2_ [kPa]^[b]^	*E*_3_ [kPa]^[b]^	*U*_T_ [MJ m^−3^]^[c]^
ISPU	2	1980±230	86.2±8.4	3.5±0.7	0.6±0.1	7.1±0.6	555.2±100.3
	10	1466±81	75.1±3.6	7.5±0.4	1.0±0.1	7.2±0.4	407.3±27.9
	100	1358±67	117.4±11.4	48.0±3.7	2.2±0.2	10.0±0.6	567.8±76.0
							
IMPU	2	2188±32	54.7±6.4	13.8±1.2	0.6±0.1	3.5±0.3	449.0±38.4
	10	1806±69	63.5±2.3	44.2±7.8	0.9±0.1	4.7±0.1	445.7±38.6
	100	1814±19	81.6±3.3	84.3±9.5	1.2±0.2	5.3±0.1	±25.0

[a] Determined by uniaxial deformation of tensile bar until failure (22 °C, *n*=3, annealed at 25 °C for 7 d after pressing). [b] Modulus in each regime during deformation. Determined by the slope of the stress–strain curve in a linear segment of each regime (22 °C, *n*=3, annealed at 25 °C for 7 d after pressing). [c] Determined by integration of the area under the stress‐strain curve obtained during uniaxial deformation (22 °C, *n*=3, annealed at 25 °C for 7 d after pressing).

**Figure 3:**
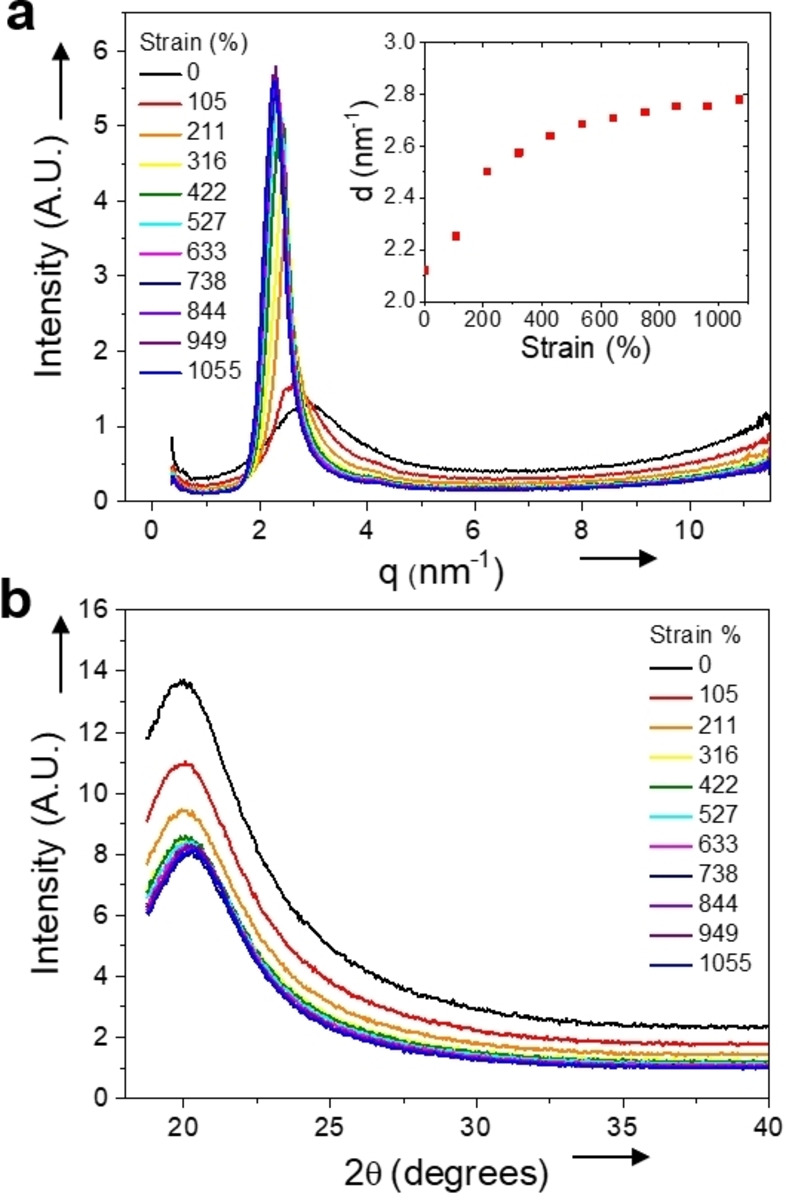
a) SAXS intensity profile of ISPU at different strains during tensile stretching at a rate of 10 mm min^−1^. b) WAXS intensity profile of ISPU at different strains during tensile stretching at a rate of 10 mm min^−1^. Inset figure shows the change in domain spacing during deformation as determined by SAXS.

ISPU was analyzed by dynamic X‐ray scattering experiments (Figure 3). The theoretical small‐angle X‐ray scattering (SAXS) profiles from ISPU and IMPU were fitted to the experimental values over a broad range of scattering vectors, *q*, with 99 % confidence bounds supporting a parametric fit. There were notable changes to the SAXS profile of ISPU during deformation (Figure 3a). Initially, a diffuse, low intensity peak was observed at 0 % strain. As the sample was stretched the scattering signal becomes stronger, narrows, and slowly shifts to lower *q* values, which suggests that progressive chain alignment occurs during deformation. This is consistent with the notable strain‐hardening profile in the tensile analysis (Figure 2a). The wide‐angle X‐ray scattering (WAXS) data also reveal that at 0 % strain ISPU possesses a broad signal at small scattering angles (2*θ*<30°), characteristic of an amorphous material (Figure 3b). A slight increase in the intensity of this amorphous peak is observed as the material is stretched, however no well‐resolved peaks appear at any strain. This means that there is no formation of crystalline domains, even during deformation, i.e. the strain‐hardening event is not a result of strain‐induced crystallization (Figure 3b).

To elucidate if the mechanical properties of these elastomers were a result of the hydrogen‐bonding network dynamics that we had designed into our materials, mechanical testing was conducted as a function of strain rate (Figure 2b). ISPU and IMPU samples strained quickly (100 mm min^−1^), had the highest stress at break and the highest modulus in each regime. On time scales shorter than the association time of the reversible bonds, the dynamic crosslinks act as a strong network and the material is elastic. This occurs because the relative time scale of deformation is shorter than the lifetime of the H‐bonds, thus the H‐bonds act as strong crosslinks and the elastic character of the material dominates.[^2b^, ^3a^, ^21^] Alternatively, when samples are strained slowly, dynamic rearrangement of the H‐bonds (transient crosslinks) can occur which enhances the viscous characteristics of the material. When strained at 2 mm min^−1^, ISPU and IMPU exhibited higher strains at break and a slower accumulation of stress than materials that were stretched more quickly. Notably, slow deformation rates did little to diminish the extent of strain hardening, but rather delayed its onset. As such, both materials were actually tougher when strained at 2 mm min^−1^ than at 10 mm min^−1^ and the difference in stereochemistry was notable with ISPU displaying ca. 140 % higher stress at break than IMPU when the samples were strained at 2 or 10 mm min^−1^.

**Figure 4:**
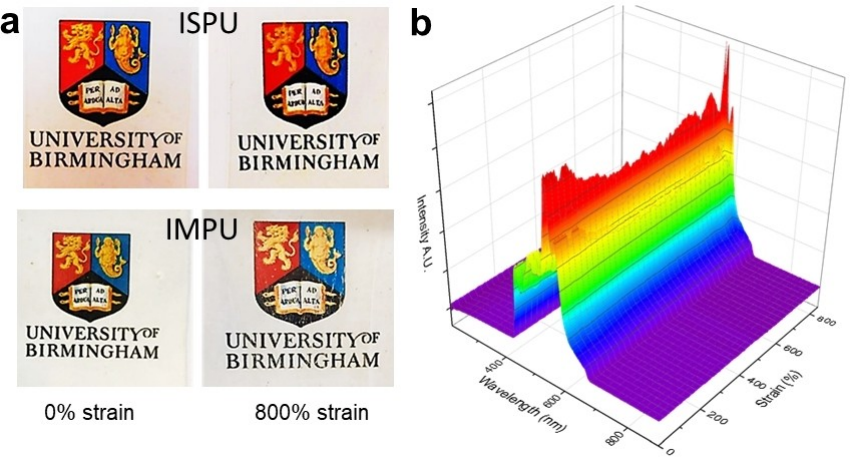
a) Images of ISPU and IMPU films prior to stretching and while being stretched to 800 % strain continue to show optical clarity (0.25 mm thick at 0 % strain). b) Optical transmittance of light through ISPU film during stretching.

During tensile testing of ISPU and IMPU, the materials remained transparent, even at large strain values (Figure 4a), which is consistent with the absence of strain‐induced crystallization being responsible for the strain‐hardening event as this would change the opacity of the material. To experimentally quantify this observation, a simple experiment was designed in which a white‐light source was passed through the material during deformation, and the intensity of the transmitted light was recorded using a spectrophotometer positioned behind the sample. When the material was subjected to tensile stress, only a slight decrease in light intensity was observed across a range from 200 % to 800 % strain (Figure 4b). This was compared to high‐density polyethylene (HDPE), a conventional synthetic material that is well‐known to undergo strain‐induced crystallization (Figure S25). In this case, a substantial decrease in optical transparency was observed as the HDPE sample was stretched to 600 % strain.

**Figure 5:**
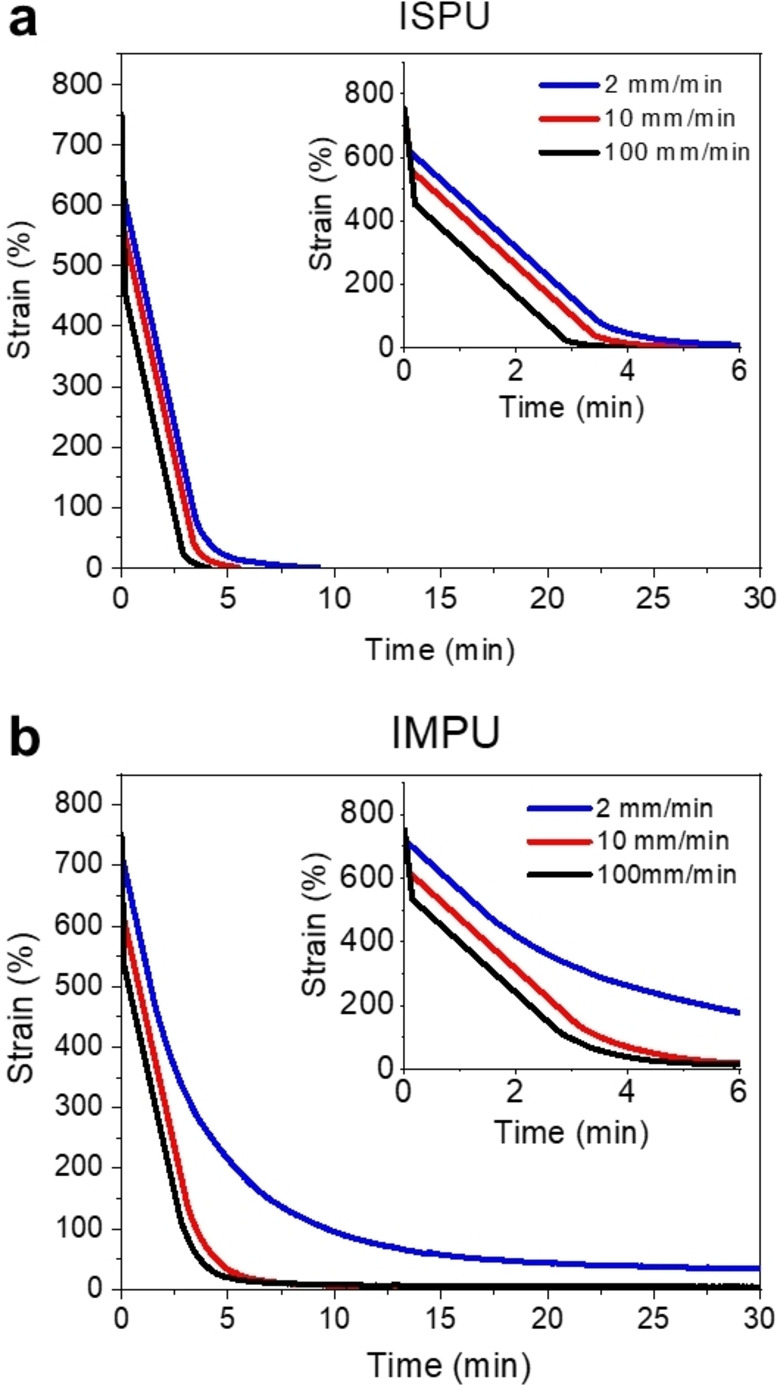
Comparison of zero‐force stress recovery after tensile stretching to 750 % as a function of strain rate in a) ISPU (Inset data between 0 and 6 minutes) and b) IMPU (22 °C, samples annealed at 25 °C for 7 d after pressing).

The stereochemical differences in the polymers’ behavior were even more pronounced during the recovery of each material from strain (Figure 5). The elastic recoveries of ISPU and IMPU were monitored as a function of stress versus time by stretching each sample to 750 % strain at different rates and allowing each to recover without a predetermined rate by maintaining zero force. Elastic recovery was affected by the deformation rate, as expected from previous reports that show elastomers based on reversible coordination systems undergo unique, time‐dependent recovery from deformation.^[22]^ Additionally, the stereochemistry of the isohexide unit in the polymer backbone played a significant role in the rate of recovery. Overall, IMPU recovered much more slowly than ISPU, particularly in the last stage of recovery. Indeed, when deformed at 2 mm min^−1^, IMPU did not recover fully. However, it was qualitatively observed that heating the deformed IMPU sample enabled complete recovery. Previous studies have reported that this phenomenon is a consequence of competition between thermodynamics favoring return to an equilibrium state and the temporary reformation of bonds in the transient network, and attribute the full recovery to the disruption of the reformed bonding network at elevated temperatures.^[22a]^

**Figure 6:**
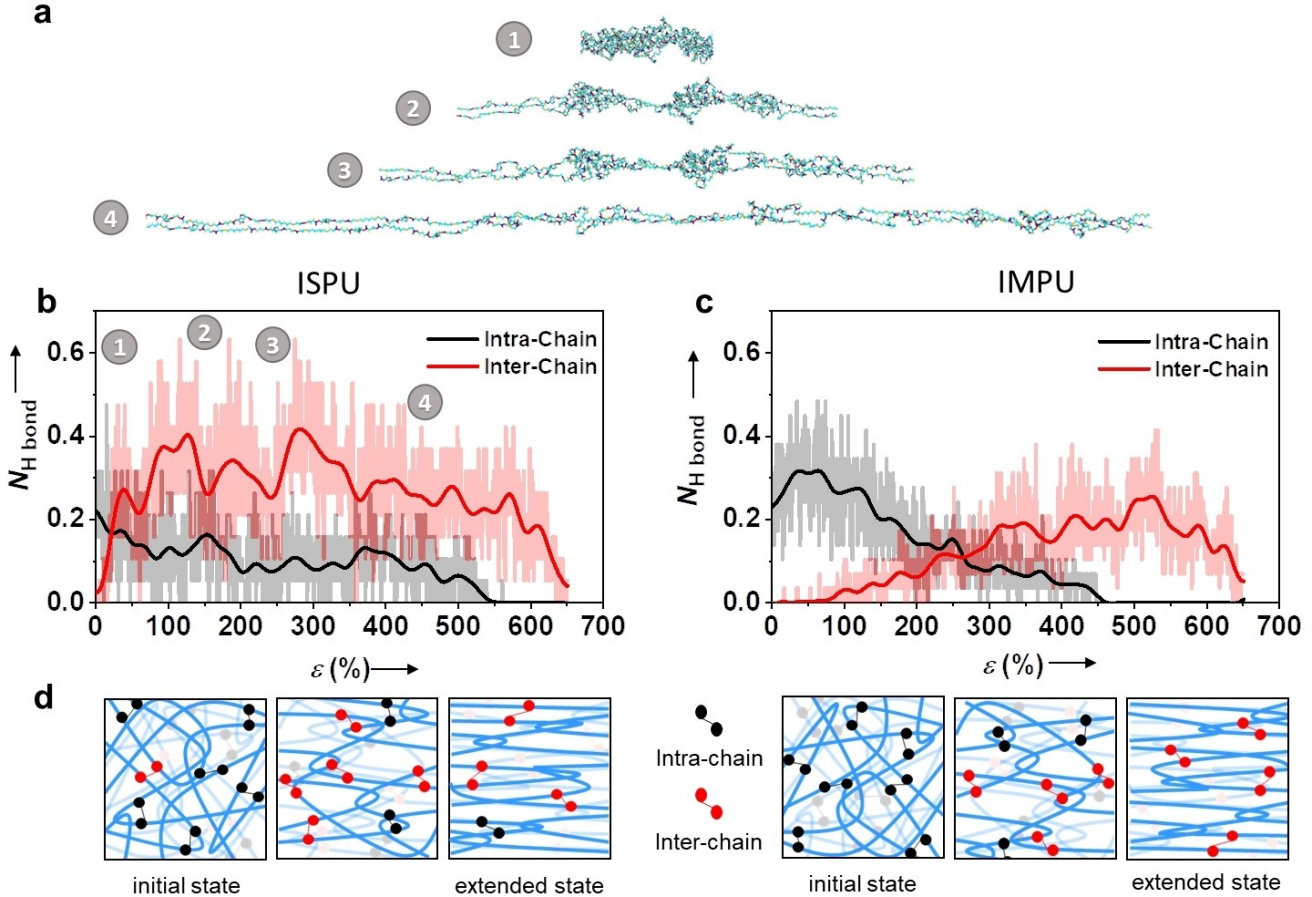
a) Simulation snapshots of ISPU chain deformations. Numbers associated with each snapshot correspond to a specific strain percent, shown in panel b. Evolution of the average number of inter‐chain *N*_inter_ and intra‐chain *N*_intra_ H‐bonds as a function of strain in b) ISPU and c) IMPU. d) Schematics of ISPU (left) and IMPU (right) chains at different strains comparing hydrogen bond evolution, suggested by data from scattering experiments and simulations.

To fully elucidate the mechanisms behind the unique properties of these materials, including how the stereochemical differences affect strain recovery, we performed atomistic molecular dynamics simulations of ISPU and IMPU chains (see Supporting Information for details). The simulations showed that with increasing deformation of the chain backbone, alignment increases (Figure 6a). Therefore, to accommodate such conformational changes and to release local chain stress, the transient network of H‐bonds is transformed as well. In particular, we observed evolution of the hydrogen‐bonding network in the materials such that destruction of the intra‐chain H‐bonds was accompanied by theformation of new inter‐chain H‐bonds (Figure 6b, c). Note that such H‐bond rearrangement also provides a dissipation channel for deformation energy. Both materials showed a transition from a transient network of intra‐ to inter‐chain H‐bonds upon elongation. However, the rate of this transition varied with the stereochemistry of the materials. ISPU showed a rapid transition from intramolecular to intermolecular bonding in the initial stages of deformation. After this initial transition, these bonds plateaued until intramolecular bonding was eliminated entirely at high strain.

Alternatively, IMPU experienced a slow loss of intramolecular bonding that corresponded with a similar increase in the number of intermolecular bonds. These differences in the materials based on stereochemistry of the isohexide unit most likely result from the bowl‐like structure that directs the hydrogen‐bonding groups within the materials as formed. We also investigated the hydrogen‐bonding of ISPU using FTIR analysis and found that the FTIR spectra of ISPU at 0 % elongation (relaxed) and 1000 % elongation (stretched) were nearly indistinguishable (Figure S30). Significant hydrogen‐bonding was apparent in both states as evidenced by a sharp N−H signal at 3340 cm^−1^ and a sharp C=O signal around 1710 cm^−1^.^[23]^

Again, the stereochemistry of the isohexide unit is clearly responsible for differences between material behaviors. The computational investigations and scattering studies suggest that intramolecular hydrogen bonds are more easily reformed in ISPU than in IMPU (Figure 6b, c, Figure S28). This is consistent with the experimental conclusion that ISPU is a stronger material than IMPU, because the reformation of bonds enables more continuous dissipation of energy during deformation. Additionally, slow rates of extension allow for localized relaxation, which facilitates the reformation of intramolecular bonds. This resulted in ISPU exhibiting a superior strength and elasticity when stretched at 2 mm min^−1^ than when stretched at 10 mm min^−1^. On the other hand, extended chain conformations appeared to restrict the reformation of intramolecular bonds in IMPU. This is evidenced by the slow recovery of IMPU relative to ISPU. Since intramolecular H‐bonds cannot be readily reformed when the IMPU chains are extended, intermolecular bonds are more likely to reform during elastic recovery. This effectively cross‐links the network more densely and hinders complete recovery to an equilibrium state (Figure 6d).

**Figure 7:**
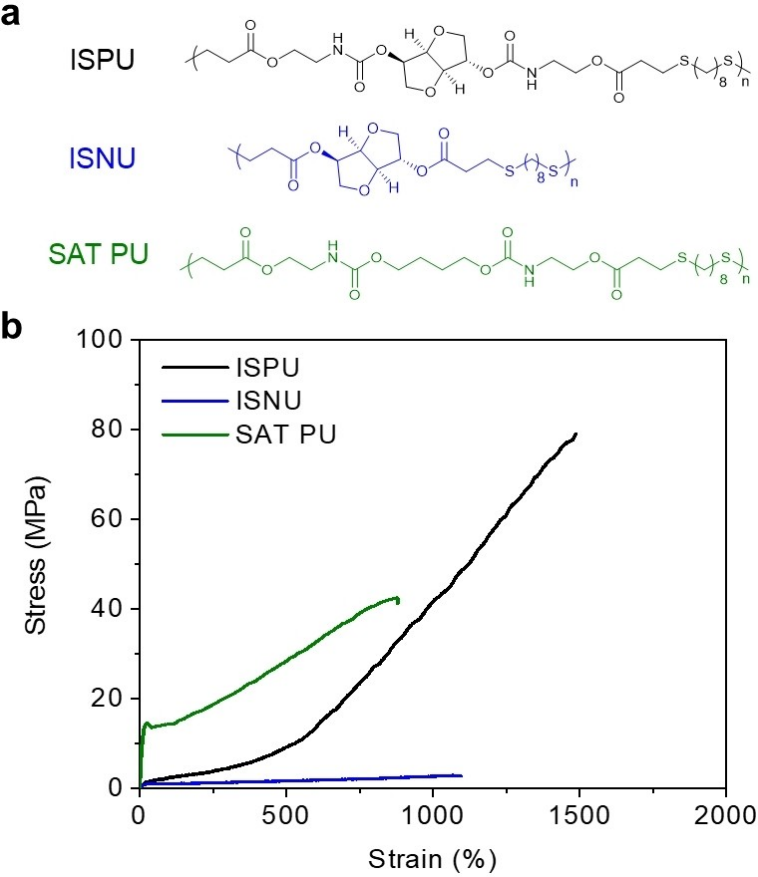
a) Scheme showing structures of ISPU, ISNU, SAT‐PU. b) Representative stress vs. strain curves obtained by tensile testing of ISPU, ISNU, and SAT‐PU films, demonstrating that the mechanical performance of ISPU is derived from the combination of the urethane groups and the sugar units. (22 °C, *n*=3, strained at 10 mm min^−1^, annealed at 25 °C for 7 d after pressing).

The optical clarity and scattering experiments lend credence to the computational observations that the strain hardening is caused by a transition from intramolecular to intermolecular hydrogen‐bonding during elongation. Furthermore, the stereochemical differences in the isohexide units that dictate the fundamental behavior of the bulk materials indicated that both the rigid isohexide and adjacent hydrogen‐bonding group(s) were required to elicit the observed behavior. To further probe this using experimental methods, two additional polymers were synthesized, as described above, using different monomers. One contained the isohexide but not urethane groups while the other contained urethane groups but not the isohexide (Figure 7a). The first was a saturated polyurethane (SAT‐PU) in which the sugar unit was replaced with a four‐carbon alkyl chain. Thermal analysis of SAT‐PU by DSC clearly revealed a semi‐crystalline structure, as evidenced by the presence of distinct crystallization and melting transitions (Figure S19). The second polymer was a non‐urethane isosorbide‐based material (ISNU), in which the urethane group was replaced with an ester. Thermal analysis of ISNU also showed that it also undergoes a melt transition, which suggests that it possesses a semi‐crystalline structure (Table 2).

**Table 2:** Characterization of ISPU compared to non‐urethane and saturated urethane analogues.

Polymer	*M*_w_^[a]^	*Đ_M_* ^[a]^	*T*_g_ [°C]^[b]^	*T*_m_ [°C]^[b]^	*T*_c_ [°C]^[b]^		*T*_onset_ [°C]^[c]^	Stress at Yield [MPa]^[d]^	Strain at Break [%]^[d]^	Stress at Break [MPa]^[d]^	*E* [MPa]^[d]^	*U_T_* [MJ m^−3^]^[d]^
ISPU	109.5	11.08	13	–	–		302	–	1466±81	75.1±3.6	7.5±0.4	407.3±27.9
ISNU	136.1	6.71	−15	48	–		347	–	1037±33	1.01±0.1	6.86±1.33	16.8±3.01
SAT‐PU	139.4	4.71	−24	96	60		301	14.8±0.3	874±13	43.2±0.7	158±2.8	240±5.9

[a] Determined by SEC in DMF. [b] Determined by DSC. [c] Determined by TGA. [d] Determined by uniaxial extension until failure.

Uniaxial tensile testing showed that ISNU and SAT‐PU had ultimate tensile strengths of 1.01±0.1 MPa and 43.2±0.7 MPa and elongations at break of 1037±33 % and 874±13 %, respectively (Figure 7b). The SAT‐PU material had a yield point at low strain that was followed by strain hardening until failure. However, the extent of hardening was notably less significant than in ISPU or IMPU, which resulted in lower tensile strength and elongation at break. In contrast, the non‐urethane analogue (ISNU) was extremely soft. The tensile strength of this material could be improved by annealing for longer durations (Figure S23), but this only increased the stress at failure to an average of 10.8 MPa, well short of the ISPU material strength (Table S2). Dynamic WAXS and SAXS analyses of both ISNU and SAT‐PU showed peaks indicative of crystallization (Figure S26, 27). These results demonstrate that both the rigid sugar moiety and the strong hydrogen‐bonding groups in the backbone are necessary to achieve significant strain hardening without crystallization, confirming the results from simulations.

## Conclusion

Combining both strength and flexibility in elastomer systems is conventionally achieved by crosslinking polymer networks or by creating phase‐separated thermoplastic elastomer structures from multi‐ or tri‐block copolymers with contrasting properties. However, by placing a rigid‐ring structure adjacent to hydrogen‐bonding units, we have shown that it is possible to create an alternating polymer that imparts both the toughness that might be expected from rigid, glassy units and the strength and flexibility that are common in other polyurethane materials. Moreover, the subtle differences in stereochemistry between the isosorbide‐ and isomannide‐derived materials lead to distinct behavioral differences that result from dynamic supramolecular transitions between intra‐ and inter‐molecular hydrogen bonding in the transient network. Here, the slightly more disordered polymer chains in the bulk that result from the regiochemical irregularity of the isosorbide stereochemistry result in the isosorbide materials displaying higher strength and faster elastic recovery. The latter observation reveals the critical role that stereochemistry plays in the direction of the hydrogen‐bonding network as ISPU was more readily able to reform intramolecular hydrogen bonds in an extended chain conformation than IMPU. Importantly, the hydrogen‐bonding network allows the formation of strong and flexible materials that display significant strain hardening —without strain‐induced crystallization or the use of composite fillers. In contrast to the majority of thermoplastic elastomers, this hydrogen‐bonding mechanical property control provides dynamic but reversible crosslinks that impart high optical clarity to the materials.

1

## Supporting information

As a service to our authors and readers, this journal provides supporting information supplied by the authors. Such materials are peer reviewed and may be re‐organized for online delivery, but are not copy‐edited or typeset. Technical support issues arising from supporting information (other than missing files) should be addressed to the authors.

Supporting Information

## Data Availability

The data that support the findings of this study are available from the corresponding author upon reasonable request.
