# Supplementary material for: Ultra‐Tough Elastomers from Stereochemistry‐Directed Hydrogen Bonding in Isosorbide‐Based Polymers
Source: Angew Chem Int Ed Engl. 2022 Mar 4;61(17):e202115904. doi: 10.1002/anie.202115904 (PMC9311410; doi:10.1002/anie.202115904)
Supplement: Supplementary file 1 — Supporting Information [file ANIE-61-0-s001.pdf]

Angewandte Chemie
Eine Zeitschrift der Gesellschaft Deutscher Chemiker

Supporting Information

Ultra-Tough Elastomers from Stereochemistry-Directed Hydrogen Bonding in Isosorbide-Based Polymers

S. R. Petersen, H. Prydderch, J. C. Worch, C. J. Stubbs, Z. Wang, J. Yu, M. C. Arno, A. V. Dobrynin, M. L. Becker*, A. P. Dove**

Supporting Information
©Wiley-VCH 2021
69451 Weinheim, Germany

Ultra-Tough Elastomers from Stereochemistry-Directed Hydrogen Bonding in Isosorbide-Based Polymers

Shannon R. Petersen, [a]† Hannah Prydderch,[b]† Joshua C. Worch,[b] Connor J. Stubbs,[b] Zilu Wang,[c] Jiayi Yu,[a] Maria C. Arno,[b] Andrey V. Dobrynin,[c] Matthew L. Becker,*[d] and Andrew P. Dove*[b]

[a] Dr. S. R. Petersen, Dr. J. Yu
Department of Polymer Science
The University of Akron
Akron, OH 44224, United States of America

[b] Dr. H. Pydderch, Dr. J. C. Worch, C. Stubbs, Dr. M. C. Arno, Prof. A. P. Dove
School of Chemistry
University of Birmingham
Birmingham B15 2TT, United Kingdom
E-mail:

[c] Dr. Z. Wang, Prof. A. V. Dobrynin
Department of Chemistry
University of North Carolina Chapel Hill
Chapel Hill, NC, 27599, United States of America

[d] Prof. M. L. Becker
Departments of Chemistry, Mechanical Engineering and Materials Science, Biomedical Engineering and Orthopaedic Surgery
Duke University
Durham, NC, 27708, United States of America
E-mail: matthew.l.becker@duke.edu

DOI: 10.1002/anie.2021XXXXX

Table of Contents

Table of Contents 2
^{1}H AND ^{13}C NMR SPECTRA 9
Isosorbide Diacrylate Urethane (ISDAU) 9
Isomannide Diacrylate Urethane (IMDAU) 10
Isosorbide Diacrylate Ester (ISDE) 11
1,4-Butanediol Diacrylate Urethane (BDDU) 12
1,8-Octanedithiol Isosorbide Polyurethane (C8 ISPU) 13
1,8-Octanedithiol Isomannide Polyurethane (C8 IMPU) 14
1,8-Octanedithiol Isosorbide polyester (C8 ISNU) 15
1-Octanedithiol-1,4-butanediol polyurethane (C8 SAT PU) 16
SIZE-EXCLUSION CHROMATOGRAPHY 17
RHEOLOGY 18
DIFFERENTIAL SCANNING CALORIMETRY 19
THERMOGRAVIMETRIC ANALYSIS 20
MECHANICAL CHARACTERIZATION 21
OPTICAL TRANSPARENCY 23
SAX and WAX 24
SIMULATION DETAILS 26
REFERENCES 29

EXPERIMENTAL

Materials

All reagents were purchased from Merck (Sigma Aldrich) unless noted and were used without further purification unless detailed. 1,8-Octanedithiol (≥ 97%) was distilled prior to use and stored under an inert atmosphere. Dimethylphenyl phosphine (99%) was purchased in an ampoule and stored under an inert atmosphere once opened. Isosorbide was recrystallized from ethyl acetate prior to use. High density polyethylene (HDPE) was obtained in pellet form from Alfa Aesar (Lot: S02D047, density = 0.95 g/mL). Nylon 6 was purchased in pellet form from Sigma-Aldrich (Lot: MKCB9179, density = 1.084 g/mL).

Preparation of Isosorbide Diacrylate Urethane (ISDAU)[29]

2-isocyanatoethyl acrylate (20.7 mL, 166 mmol) and dibutyltin dilaurate (81.0 µL, 136 µmol) were added to a solution of isosorbide (10.00 g, 146 mmol) in dry THF (60 mL) while stirring. The reaction mixture was stirred overnight at ambient temperature. The product was isolated via precipitation by removing the majority of the THF *in vacuo* followed by the addition of diethyl ether (100 mL). The resulting solid was collected by vacuum filtration and subsequently re-crystallized from toluene:isopropyl alcohol (80:20) to yield ISDAU as a white crystalline solid (23.3 g, 54.4 mmol), 80% yield. ^{1}H NMR (400 MHz, $CDCl_3$) δ 6.43 (d, *J* = 17.3 Hz, 2H), 6.12 (dd, *J* = 17.3, 10.4 Hz, 2H), 5.87 (d, *J* = 10.4 Hz, 2H), 5.12 (m, 4H), 4.76 (t, *J* = 4.7 Hz, 1H), 4.49 (d, *J* = 4.4 Hz, 1H), 4.24 (t, *J* = 5.3 Hz, 4H), 4.04 – 3.92 (m, 3H), 3.71 (m, 1H), 3.50 (m, 4H). ^{13}C NMR (101 MHz, $CDCl_3$) δ 166.16, 166.12, 155.60, 155.26, 131.70, 131.65, 128.03, 85.95, 81.13, 78.75, 77.48, 77.16, 76.84, 74.57, 73.90, 70.10, 63.54, 63.50, 40.38. HRMS (ESI+, m/z) calculated for $C_{18}H_{24}N_2O_{10}Na$, requires 451.1329; found 451.1330.

Preparation of Isomannide Diacrylate Urethane (IMDAU)[29]

2-isocyanatoethyl acrylate (20.7 mL, 166 mmol) and dibutyltin dilaurate (81.0 µL, 136 µmol) were added to a solution of isomannide (10.00 g, 146 mmol) in dry THF (60 mL) while stirring. The reaction mixture was stirred overnight at ambient temperature. The product was isolated via precipitation by removing the majority of the THF *in vacuo* followed by the addition of diethyl ether (100 mL). The resulting solid was collected by vacuum filtration and subsequently recrystallized from toluene:isopropyl alcohol (80:20) to yield IMDAU as a white crystalline solid (22.1 g, 51.7 mmol), 76% yield. ^{1}H NMR (400 MHz, $CDCl_3$) δ 6.43 (d, *J* = 17.3 Hz, 2H), 6.12 (dd, *J* = 17.3, 10.4 Hz, 2H), 5.87 (d, *J* = 10.4 Hz, 2H), 5.16 (m, 2H), 5.10 (m, 2H), 4.67 (d, *J* = 4.2 Hz, 2H), 4.25 (t, *J* = 5.3 Hz, 4H), 4.07 (dd, *J* = 9.2, 6.5 Hz, 2H), 3.85 – 3.68 (m, 2H), 3.51 (m, 4H). ^{13}C NMR (101 MHz, $CDCl_3$) δ 166.14, 155.56, 131.70, 128.03, 80.95, 77.16, 74.42, 70.65, 63.57, 40.42. HRMS (ESI+, m/z) calculated for $C_{18}H_{24}N_2O_{10}Na$, requires 451.1329; found 451.1331.

Preparation of 1,4-Butanediol Diacrylate Urethane (BDDU)

A single neck 100 mL round bottom flask was charged with 1,4-butanediol (4.50 g, 50 mmol, 1.00 equiv) dissolved in THF (26 mL) and cooled to 0 °C with an ice bath. The solution was stirred and 2-isocyanatoethyl acrylate (14.80 g, 105 mmol, 2.10 equiv) was charged via syringe into the round bottom flask. DBTDL (60 µL, 0.2 mol%) was added to the solution and left to stir overnight *ca*. 16 h. During this time a white precipitate was formed. The remaining solvent was then removed under vacuum. The resulting white powder was purified by recrystallization from EtOAc/Hexanes to afford BDDU as a white crystalline solid (15.4 g, 83%). ^{1}H NMR (300 MHz,

$CDCl_3$) δ 6.43 (d, *J* = 17.3 Hz, 2H), 6.13 (dd, *J* = 17.3, 10.4 Hz, 2H), 5.86 (d, *J* = 10.4 Hz, 2H), 5.00 (s, 1H), 4.24 (t, *J* = 5.3 Hz, 4H), 4.10 (m, 4H), 3.48 (q, *J* = 5.6 Hz, 4H), 1.69 (d, *J* = 5.8 Hz, 4H). ^{13}C NMR (101 MHz, $CDCl_3$) δ 166.20, 156.65, 131.59, 128.10, 77.48, 77.16, 76.84, 64.74, 63.72, 40.23, 25.73. HRMS (ESI-TOF) (m/z): $[M + H]^+$ calculated for $C_{16}H_{24}N_2O_8Na$, requires 395.1430; found 395.1432.

Preparation of Isosorbide Diacrylate Ester (ISDE)[41]

A flask was charged with isosorbide (8.00 g, 54.7 mmol) and dry CH_2Cl_2 (100 mL) under N_2. Dry NEt_3 (17.6 mL, 126 mmol) was added in one portion and the reaction was cooled to 0 °C. Acryloyl chloride (9.78 mL, 120 mmol) in dry CH_2Cl_2 (50 mL) was added dropwise to the reaction over 2 h to control any exotherm. On complete addition the reaction was stirred overnight at ambient temperature. Reaction completion was confirmed by thin layer chromatography (50:50 ethyl acetate:hexane) and the precipitated triethylammonium chloride was removed by filtration. The filtrate was washed with 1N HCl solution (2 × 50 mL), saturated sodium bicarbonate solution (2 × 50 mL), saturated sodium chloride solution (100 mL) and dried over $NaSO_4$. Volatiles were removed *in vacuo* to yield the crude compound as an orange solid. Purification by silica-gel column chromatography (50:50 ethyl acetate:hexane) furnished a white crystalline solid (9.36 g, 37.1 mmol), 67% yield. ^1H NMR (400 MHz, $CDCl_3$) δ 6.52 – 6.37 (m, 2H), 6.29 – 6.00 (m, 2H), 5.94 – 5.81 (m, 2H), 5.31 – 5.18 (m, 2H), 4.94 – 4.84 (m, 1H), 4.54 (d, *J* = 4.6 Hz, 1H), 4.06 – 3.94 (m, 3H), 3.91 – 3.80 (m, 1H). ^1H NMR data is in agreement with the literature.[41] ^{13}C NMR (101 MHz, $CDCl_3$) δ 165.54, 165.22, 132.00, 131.92, 127.88, 127.76, 86.08, 81.00, 78.23, 74.19, 73.57, 70.49. HRMS (ESI+, m/z) calculated for $C_{12}H_{14}O_6Na$, requires 277.0688; found 277.0689.

Preparation of 1,8-Octanedithiol Isosorbide Polyurethane (ISPU)

To a stirred solution of ISDAU (4.63 g, 10.8 mmol) in chloroform was added 1,8-octanedithiol (1.93 g, 10.8 mmol) followed by a catalytic amount of dimethylphenyl phosphine (30.7 µL, 0.216 mmol). The reaction mixture was stirred at 50 °C for 16 h. After 16 h, the reaction mixture was cooled to ambient temperature and diluted with chloroform (50 mL). The polymer was precipitated from solution by dropwise addition into diethyl ether (1000 mL) whilst stirring to give a white rubbery solid which was further washed with diethyl ether (500 mL). The polymer was dried in a vacuum oven for 5 h at 90 °C to give an off-white solid (6.13 g), 93% yield. ^1H NMR (400 MHz, $CDCl_3$) δ 5.33 (s, 1H), 5.26 (s, 1H), 5.14 – 5.05 (m, 2H), 4.76 (t, *J* = 4.8 Hz, 1H), 4.49 (d, *J* = 4.5 Hz, 1H), 4.22 – 4.12 (m, 4H), 4.03 – 3.91 (m, 3H), 3.71 (dd, *J* = 9.6, 6.2 Hz, 1H), 3.45 (dq, *J* = 11.7, 5.9 Hz, 4H), 2.77 (t, *J* = 7.2 Hz, 4H), 2.62 (t, *J* = 7.2 Hz, 4H), 2.52 (t, *J* = 7.4 Hz, 4H), 1.63 – 1.51 (m, 4H), 1.42 – 1.25 (m, 8H). ^{13}C NMR (101 MHz, $CDCl_3$) δ 171.97, 155.67, 155.34, 86.01, 81.13, 78.74, 77.48, 77.16, 76.84, 74.55, 73.86, 70.17, 63.65, 63.60, 40.28, 34.80, 32.24, 29.59, 29.18, 28.86, 27.12. M_w = 56.5 kg mol^{-1}. $Đ_M$ = 7.86.

Preparation of 1,8-Octanedithiol Isomannide Polyurethane (IMPU)

To a stirred solution of IMDAU (4.60 g, 10.8 mmol) in chloroform was added 1,8-octanedithiol (1.93 g, 10.8 mmol) followed by a catalytic amount of dimethylphenyl phosphine (30.7 µL, 0.216 mmol). The reaction mixture was stirred at 50 °C for 16 h. After 16 h, the reaction mixture was cooled to ambient temperature and diluted with chloroform (50 mL). The polymer was precipitated from solution by dropwise addition into diethyl ether (1000 mL) whilst stirring to give a white rubbery solid which was further washed with diethyl ether (500 mL). The polymer was dried in a vacuum oven for 5 h at 90 °C to give an off-white solid (5.73 g), 87% yield. ^1H NMR (400 MHz, $CDCl_3$) δ 5.34 (s, 2H), 5.09 (s, 2H), 4.77 – 4.57 (m, 2H), 4.28 – 4.12 (m, 4H), 4.06 (dd, *J* = 9.1, 6.6 Hz, 2H), 3.89 – 3.71 (m, 2H), 3.46 (m, 4H), 2.77 (t, *J* = 7.2, 4H), 2.62 (t, *J* = 7.2,

4H), 2.57 – 2.48 (m, 4H), 1.62 – 1.51 (m, 4H), 1.44 – 1.24 (m, 8H). ^{13}C NMR (101 MHz, $CDCl_3$) δ 172.01, 155.64, 80.90, 77.16, 74.40, 70.57, 63.63, 40.29, 34.79, 32.24, 29.59, 29.18, 28.87, 27.04. M_w = 109.5 kg mol^{-1}. $Đ_M$ = 11.08.

Preparation of 1,8-Octanedithiol Isosorbide Polyester (ISNU)

To a stirred solution of IDAE (1.97 g, 7.81 mmol) in *N*,*N*-dimethylformamide (30 mL) was added 1,8-octanedithiol (1.39 g, 7.81 mmol) followed by a catalytic amount of dimethylphenyl phosphine (22.2 µL, 0.156 mmol). The reaction mixture was stirred at 50 °C for 16 h. After 16 h, the reaction mixture was cooled to room temperature and diluted with chloroform (30 mL). The polymer was precipitated from solution by dropwise addition into diethyl ether (500 mL) whilst stirring to give a white rubbery solid which was further washed with diethyl ether (2 x 500 mL). The polymer was dried in a vacuum oven for 5 h at 90 °C to give a white solid (2.81 g), 84% yield. (400 MHz, $CDCl_3$) δ 5.24 – 5.21 (m, 1H), 5.20 – 5.14 (m, 1H), 4.87 – 4.81 (m, 1H), 4.49 (d, *J* = 4.8 Hz, 1H), 3.97 (d, *J* = 2.4 Hz, 2H), 3.94 (dd, *J* = 9.9, 5.9 Hz, 1H), 3.81 (dd, *J* = 9.9, 5.2 Hz, 1H), 2.84 – 2.71 (m, 4H), 2.69 – 2.59 (m, 4H), 2.57 – 2.46 (m, 4H), 1.61 – 1.52 (m, 4H), 1.40 – 1.32 (m, 4H), 1.31 – 1.24 (m, 4H). ^{13}C NMR (101 MHz, $CDCl_3$) δ 171.51, 171.18, 86.01, 80.88, 78.30, 74.24, 73.50, 70.52, 34.82, 34.71, 32.30, 32.25, 29.62, 29.23, 28.91, 26.99, 26.96. M_w = 136.1 kg mol^{-1}. $Đ_M$ = 6.71.

Polymer Film Formation

Thin polymer films were prepared using a Specac Atlas™ Manual Hydraulic Press 15T fitted with Specac heated plates. Films with a thickness of 0.5 ± 0.05 mm were prepared by melt compressing the polymers under ca. 5 kN of force at 120 °C followed by cooling to room temperature in the press whilst under compression. To ensure consistency in the film thickness a rectangular steel spacer (0.5 mm) was employed. The machine was preheated to 120 °C and then polymer was added into the 40 × 50 × 0.5 mm mold between PTFE sheeting (0.5 mm thickness) and placed into the compression machine with heated platens touching the PTFE. After 12 min of melting the polymer was compressed with 2.5 T of pressure and released five times to ensure that no bubbles were present in the films. Next, 5 T of pressure was applied for 4 min. The press was then cooled to room temperature whilst maintaining 5 T of pressure on the sample. Note: HDPE and Nylon 6 pellets were compression molded into films above their respective melt temperatures using a similar protocol. The sample was then removed from the machine and carefully removed from the mold. Visual inspection of the films was carried out to ensure no bubbles were present before use.

Nuclear Magnetic Resonance (NMR) Spectroscopy

All NMR spectra were recorded in deuterated chloroform (99.8% D) on a Bruker 400 MHz spectrometer with chemical shifts reported in parts per million (ppm) relative to the internal standard (TMS) and coupling constants (J) are reported in Hertz (Hz).

Size-Exclusion Chromatography (SEC)

Size exclusion chromatography (SEC) in DMF was performed on an Agilent 1260 Infinity II LC system equipped with a Wyatt Optilab T-rEX differential refractive index detector, an Agilent 1260 Infinity II WR diode array detector, an Agilent guard column (PLGel 5 µM, 50 × 7.5 mm) and two Agilent Mixed-C columns (PLGel 5 µM, 300 × 7.5 mm). The mobile phase was DMF (HPLC grade) containing 5 mM NH_4BF_4 at 50 °C at flow rate of 1.0 mL min^{-1}. Number average molecular weights (M_n), weight average molecular weights (M_w) and molar mass distributions

($Đ_M$ = M_w/M_n) were determined using Wyatt ASTRA v7.1.3 software against poly(methyl methacrylate) (PMMA) standards.

Thermogravimetric Analysis (TGA)

TGA was obtained using a Discovery TGA550 Auto (TA instruments). TGA thermograms were recorded under an N_2 atmosphere at a heating rate of 10 K min^{-1}, from 0 °C to 500 °C, with an average sample weight of ca. 10 mg. Aluminum pans were used for all TGA experiments. Decomposition temperatures were reported as the onset temperature of decomposition (T_{onset}).

Differential Scanning Calorimetry (DSC)

DSC was carried out using a STARe system DSC3 (Mettler Toledo, Switzerland). DSC calorigrams were recorded under N_2 purge at standard heating and cooling rates of 10 K min^{-1}, from -50 °C to 150 °C, with a sample weight of 5–10 mg in 40 uL aluminum pan. All DSC data are reported as first run data (heating cycle) unless otherwise stated. The glass transition temperatures (Tg) were determined from the minimum of the first derivative of the exotherm transition in the first heating cycle of the DSC.

Mechanical Testing

Mechanical characteristics were investigated using a Testometric MC350-5CT with a 100 or 5 kgF cell. All samples were measured using a 100 kgF cell except for C8 IS PE annealed at 25 °C for 1 week which was measured using a 5 kgF cell. Measurements of uniaxial deformation until failure and stress recovery were performed at room temperature (22 ± 1 °C) using the compression molded films cut into dumbbell-shaped samples using a custom ASTM Die D-638 Type V with a hand press. The gauge length was set at 7.1 mm and the crosshead speed set to 2 mm min^{-1}, 10 mm min^{-1}, or 100 mm min^{-1}. The sample width (ca. 1.60 mm) and thickness (ca. 0.5 mm) were measured for each individual sample before mechanical analysis was conducted. Samples were tested after annealing for 7 days at 25 °C. For uniaxial deformation experiments, a minimum of three specimens were tested for each sample with the mean average and standard deviation of the values reported herein. In the elastic recovery experiment, zero-force was maintained after uniaxial deformation to 750% strain and stress was recorded as a function of time.

Rheology

Rheology was performed on an Anton Paar MCR 302 using a PP8 geometry on discs (8 × 1 mm) cut out of homopolymer films. Temperature was controlled with a P-PTD 200/AIR Peltier and a P-PTD 200 hood. Frequency sweeps were performed at 1% strain from 0.1 to 100 rad s^{-1} at 5°C intervals (between the temperatures of 120 °C – 150 °C for ISPU and 100 °C – 130 °C for IMPU). G' and G'' were overlaid to a single spectrum at a reference temperature of 130 °C by applying a Williams-Landel-Ferry (WLF) time-temperature superposition. Molecular entanglement was extracted by fitting polydisperse double reptation theory in the REPTATE software package. Molecular weights obtained from SEC were discretized to 20 values per decade and used as theory input. The adjustable parameters in the fitting were G_e (entanglement modulus) M_e (entanglement molecular weight), τ_e (Rouse time of one entangled segment) and the value of M_0 was kept to a value of 0.001 kg·mol^{-1} as recommended.

Optical clarity experiment

A polymer film annealed for 7 days was cut into a bar (10 × 3 × 0.5 mm) and clamped into a Testometric M100-1CT fitted with a 10 kN load cell and a preload force of 0.1 N was applied. The fiber optic detector attached to the Ocean optics USB2000+ module was clamped behind the polymer bar ensuring the detector was covered by the polymer films. A white light source was produced from an Epson Powerlite projector and positioned 20 cm in front of the polymer film. The sample was elongated at a rate of 10 mm/min. The photospectrometer was set to sample at a rate of 3 times a minute until sample breakage, the sample was then removed, and a final spectrum was taken without the polymer sample between the spectrometer and the light source.

Small-angle X-ray Scattering (SAXS) and Wide-angle X-ray Scattering (WAXS)

SAXS and WAXS measurements were carried out on dumbbell-shaped samples (prepared as stated previously) at the University of Warwick using a Linkam Scientific TST250V Tensile Testing System. Samples were measured whilst under vacuum whilst the temperature was maintained at 22 °C with 10 min SAXS/WAXS collections after each 7.5 mm pull at a rate of 10 mm/min.

Simulations

Atomistic molecular dynamics simulations were performed to elucidate effect of the chain deformation on evolution of the hydrogen bonds. The General Amber Force Field[1] (GAFF) was used to model the isosorbide/isomannide-containing polyurethanes (**Figure S29**). The forces on the atoms were calculated by differentiating the potential energy of the system, which consisted of bonded (bonds, angle, dihedral, and improper potentials) interactions as well as nonbonded interactions (van de Waals and electrostatic).

$$U = \sum_{Bonds} k_r\left(r - r_{eq}\right)^2 + \sum_{Angles} k_\theta\left(\theta - \theta_{eq}\right)^2 + \sum_{Dihedrals} k_d\left[1 + \mathrm{d}\cos(n\phi)\right] + \sum_{i<j}\left(4\epsilon_{ij}\left[\left(\frac{\sigma}{r_{ij}}\right)^{12} - \left(\frac{\sigma}{r_{ij}}\right)^{6}\right] + \frac{q_i q_j}{4\pi\varepsilon_0 \varepsilon r_{ij}}\right) \quad (1)$$

where $\varepsilon_0 = 8.85 \times 10^{-12}\ F\ m^{-1}$ is dielectric permittivity of the vacuum, ε is a medium relative dielectric constant.

In GAFF, the van de Waals interactions are represented by the Lennard-Jones (LJ) potentials with parameters given for each homogeneous atomic pair (r_{ii} and ϵ_{ii}), while the parameters for heterogeneous pairs are determined as $r_{ij} = r_i + r_j$ and $\epsilon_{ij} = \sqrt{\epsilon_i \epsilon_j}$. The LJ potential is truncated at a cutoff distance of 10 A. The weighting coefficient for the 1-4 interaction was set to 0.5 for the LJ-interactions and 0.833 for the Columbic interactions. Partial charge distributions were obtained from DFT calculations using B3LYP 6-31G* (d,p) basis set for AM1 optimized structures. These simulations were performed using Gaussian 09. Note that the partial charges are averaged for atoms with the same chemical environment for simplicity (see **Figure S29**). The interaction parameters for non-bonded/bonded interactions are summarized in **Table S3**.

The information about macromolecular structure (list of bonds, angles, dihedrals, improper dihedrals) was generated by Topotools plugins in VMD.[2] Each polymer chain had 16 repeating units. The two ends of each polymer chain were connected across the periodic boundary along x direction forming a loop representing an infinitely long polymer chain. There were two identical chains in the periodic simulation box with equilibrium dimensions L_x=80 Å and L_y=L_z=40 Å. These polymer chains adopted a bundle-like conformation. The system was equilibrated for 5000 ns. After completion of the equilibration step the simulation box was deformed along x-direction with a constant strain rate of 2.5×10^{-6} fs^{-1}. During the deformation process, the atom

velocities in y and z directions were coupled to the thermostat to maintain system temperature. All simulations were performed using the following setup: PPPM[3,4] method for calculations of the electrostatic interactions with targeted accuracy 10^{-4}, vacuum dielectric constant ε = 1.0, T = 293K, Langevin thermostat (damping parameter 10 fs), and time step Δt = 0.5 fs. All simulations were performed using LAMMPS with GPU acceleration.[5]

^{1}H AND ^{13}C NMR SPECTRA

Isosorbide Diacrylate Urethane (ISDAU)

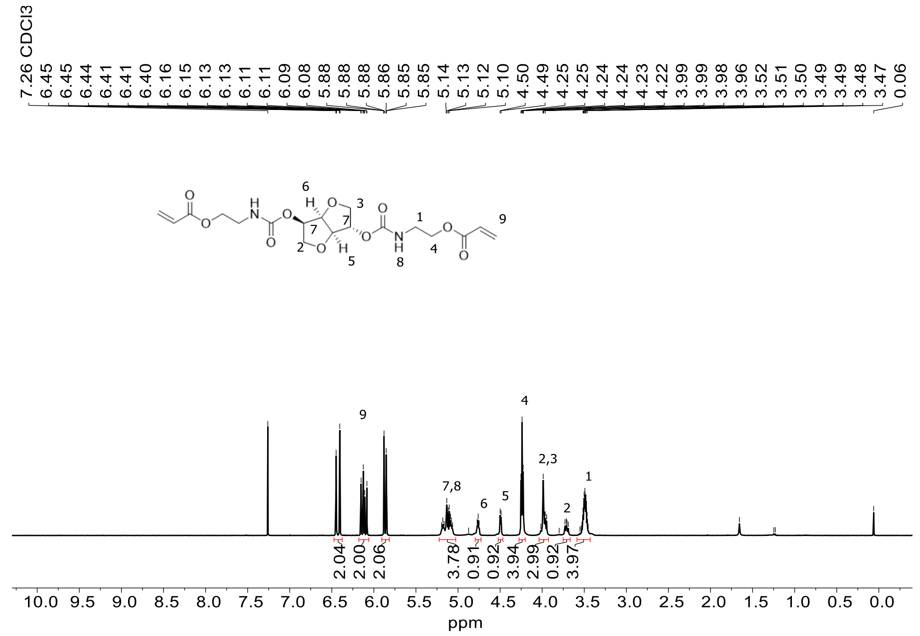

Figure S1. ^{1}H NMR spectra of isosorbide diacrylate urethane (ISDAU) ^{1}H NMR (400 MHz, $CDCl_3$) δ 6.43 (d, J = 17.3 Hz, 2H), 6.12 (dd, J = 17.3, 10.4 Hz, 2H), 5.87 (d, J = 10.4 Hz, 2H), 5.12 (m, 4H), 4.76 (t, J = 4.7 Hz, 1H), 4.49 (d, J = 4.4 Hz, 1H), 4.24 (t, J = 5.3 Hz, 4H), 4.04 – 3.92 (m, 3H), 3.71 (m, 1H), 3.50 (m, 4H).

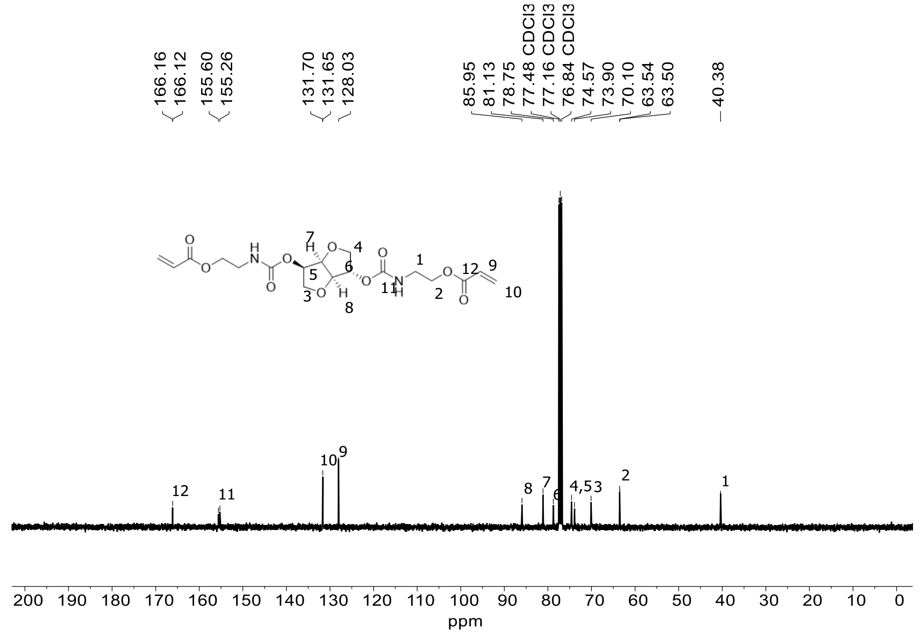

Figure S2. ^{13}C NMR spectra of isosorbide diacrylate urethane (ISDAU) (101 MHz, $CDCl_3$) ^{13}C NMR (101 MHz, $CDCl_3$) δ 166.16, 166.12, 155.60, 155.26, 131.70, 131.65, 128.03, 85.95, 81.13, 78.75, 77.48, 77.16, 76.84, 74.57, 73.90, 70.10, 63.54, 63.50, 40.38.

SUPPORTING INFORMATION

Isomannide Diacrylate Urethane (IMDAU)

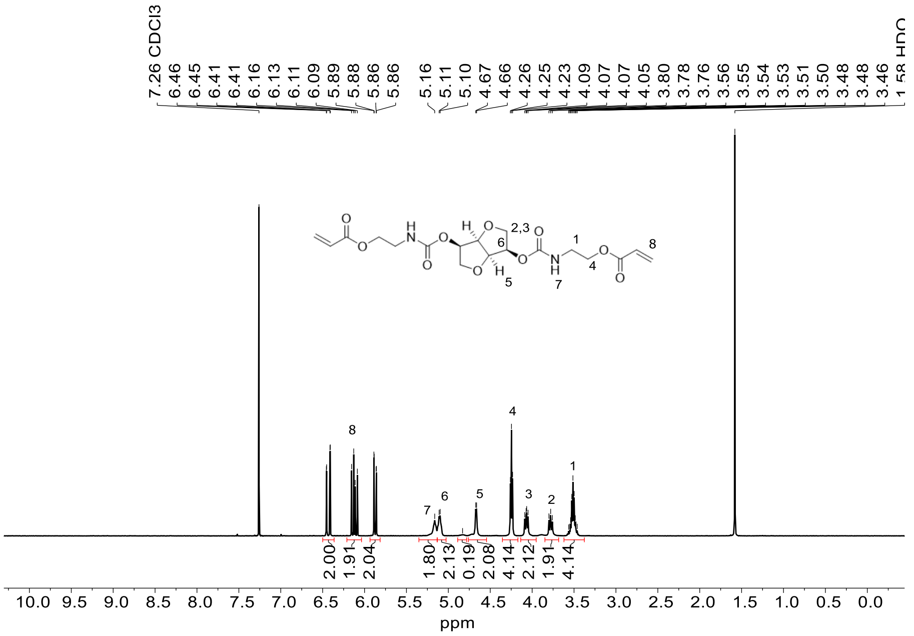

Figure S3. [1] H NMR spectra of isomannide diacrylate urethane (IMDAU) ^1H NMR (400 MHz, $CDCl_3$) δ 6.43 (d, J = 17.3 Hz, 2H), 6.12 (dd, J = 17.3, 10.4 Hz, 2H), 5.87 (d, J = 10.4 Hz, 2H), 5.16 (m, 2H), 5.10 (m, 2H), 4.67 (d, J = 4.2 Hz, 2H), 4.25 (t, J = 5.3 Hz, 4H), 4.07 (dd, J = 9.2, 6.5 Hz, 2H), 3.85 – 3.68 (m, 2H), 3.51 (m, 4H)

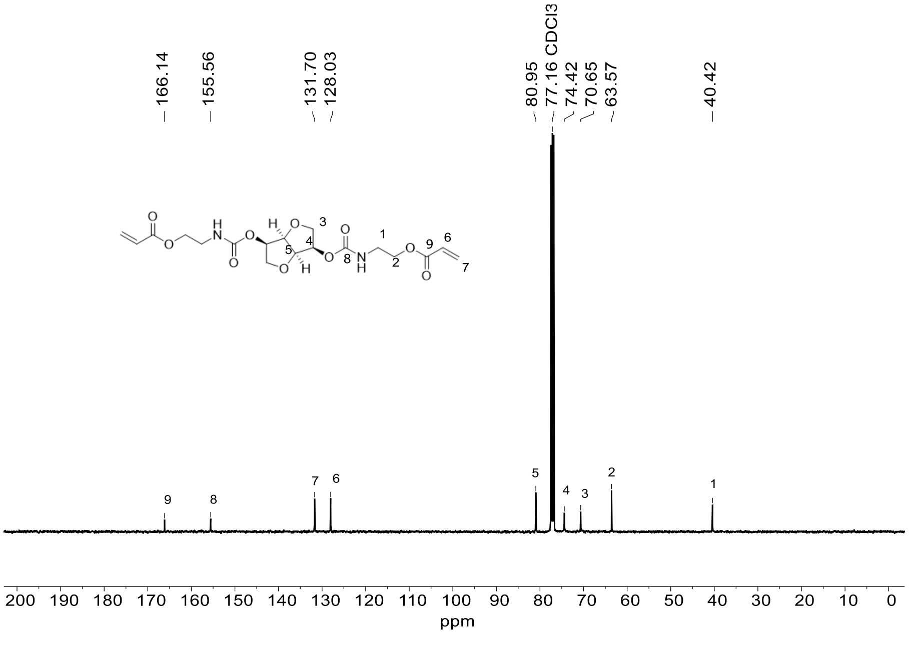

Figure S4. ^{13}C NMR spectra of isomannide diacrylate urethane (IMDAU) ^{13}C NMR (101 MHz, $CDCl_3$) δ 166.14, 155.56, 131.70, 128.03, 80.95, 77.16, 74.42, 70.65, 63.57, 40.42.

Isosorbide Diacrylate Ester (ISDE)

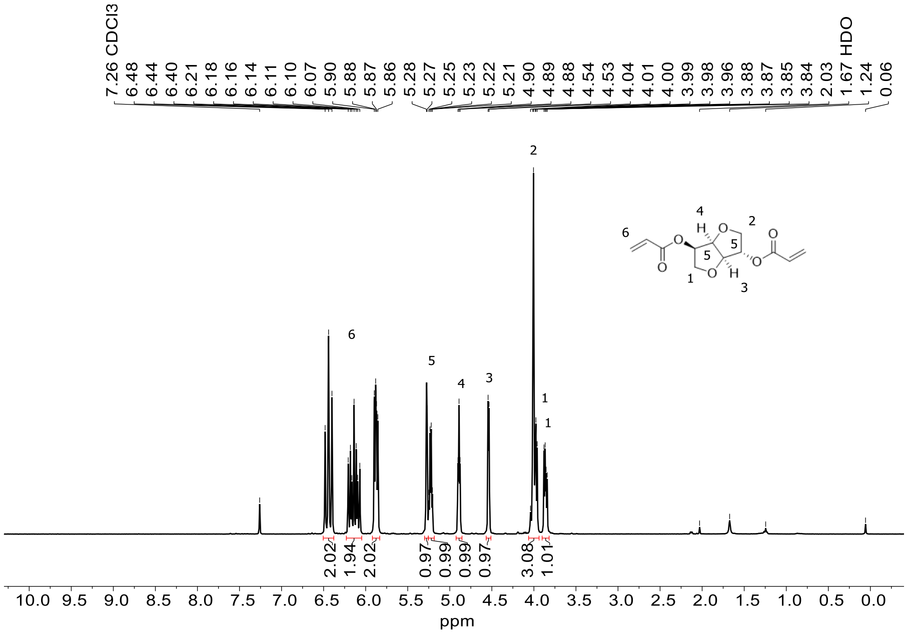

Figure S5. ^{1}H NMR spectra of isosorbide diacrylate ester (IDAE) (400 MHz, Chloroform-*d*) δ 6.52 – 6.37 (m, 2H), 6.29 – 6.00 (m, 2H), 5.94 – 5.81 (m, 2H), 5.31 – 5.18 (m, 2H), 4.94 – 4.84 (m, 1H), 4.54 (d, *J* = 4.6 Hz, 1H), 4.06 – 3.94 (m, 3H), 3.91 – 3.80 (m, 1H).

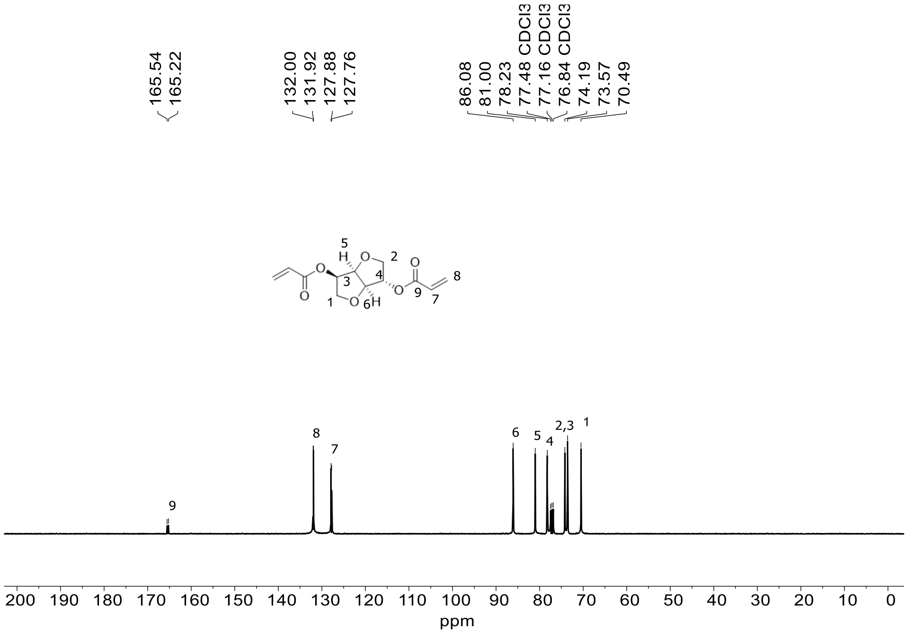

Figure S6. ^{13}C NMR spectra of isosorbide diacrylate ester (IDAE) (101 MHz, Chloroform-*d*) δ 165.54, 165.22, 132.00, 131.92, 127.88, 127.76, 86.08, 81.00, 78.23, 74.19, 73.57, 70.49.

1,4-Butanediol Diacrylate Urethane (BDDU)

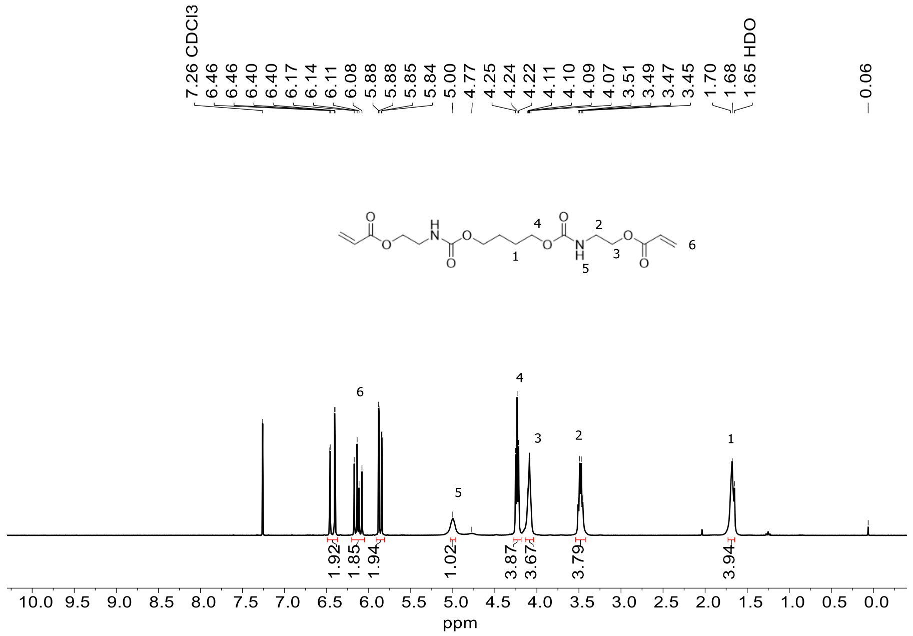

Figure S7. ^{1}H NMR spectra of 1,4-Butanediol Diacrylate Urethane (BDDU). ^{1}H NMR (300 MHz, Chloroform-*d*) δ 6.43 (d, *J* = 17.3 Hz, 2H), 6.13 (dd, *J* = 17.3, 10.4 Hz, 2H), 5.86 (d, *J* = 10.4 Hz, 2H), 5.00 (s, 1H), 4.24 (t, *J* = 5.3 Hz, 4H), 4.10 (m, 4H), 3.48 (q, *J* = 5.6 Hz, 4H), 1.69 (d, *J* = 5.8 Hz, 4H).

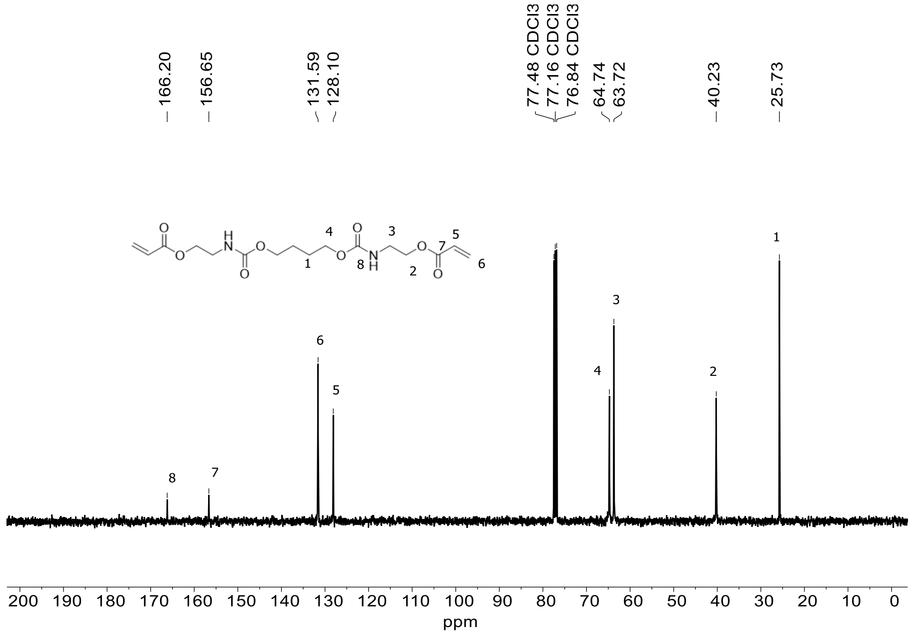

Figure S8. ^{13}C NMR spectra of 1,4-Butanediol Diacrylate Urethane (BDDU). ^{13}C NMR (101 MHz, $CDCl_3$) δ 166.20, 156.65, 131.59, 128.10, 77.48, 77.16, 76.84, 64.74, 63.72, 40.23, 25.73.

1,8-Octanedithiol Isosorbide Polyurethane (C8 ISPU)

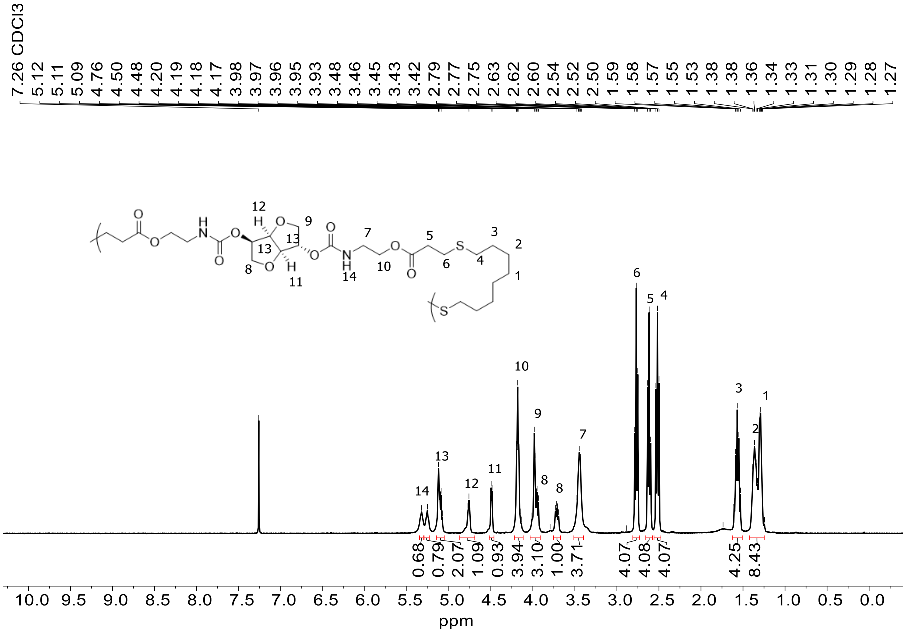

Figure S9. ^{1}H NMR spectra of 1,8-octanedithiol isosorbide polyurethane (C8 ISPU). ^{1}H NMR (400 MHz, $CDCl_3$) δ 5.33 (s, 1H), 5.26 (s, 1H), 5.14 – 5.05 (m, 2H), 4.76 (t, *J* = 4.8 Hz, 1H), 4.49 (d, *J* = 4.5 Hz, 1H), 4.22 – 4.12 (m, 4H), 4.03 – 3.91 (m, 3H), 3.71 (dd, *J* = 9.6, 6.2 Hz, 1H), 3.45 (dq, *J* = 11.7, 5.9 Hz, 4H), 2.77 (t, *J* = 7.2 Hz, 4H), 2.62 (t, *J* = 7.2 Hz, 4H), 2.52 (t, *J* = 7.4 Hz, 4H), 1.63 – 1.51 (m, 4H), 1.42 – 1.25 (m, 8H).

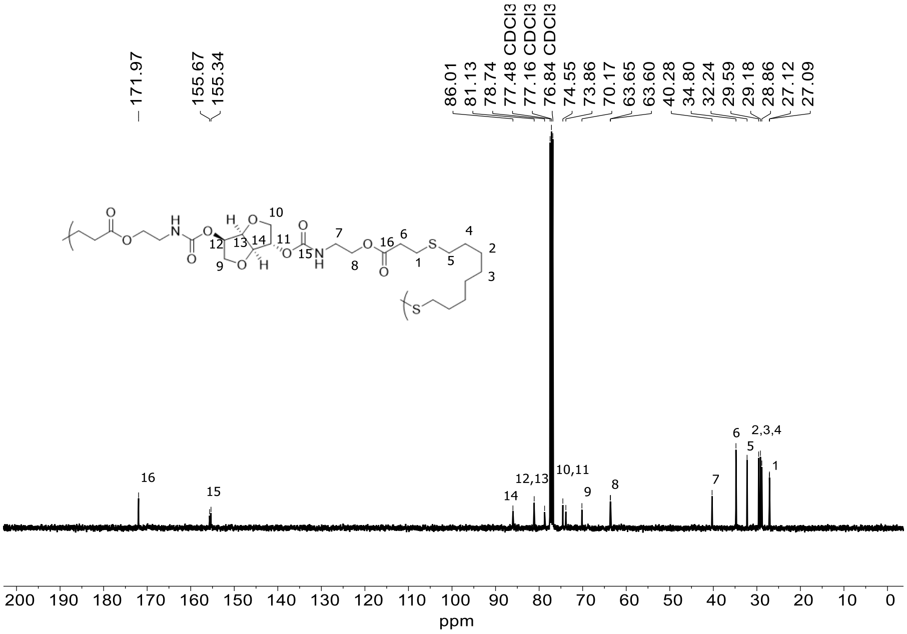

Figure S10. ^{13}C NMR spectra of 1,8-octanedithiol isosorbide Polyurethane (C8 ISPU) ^{13}C NMR (101 MHz, $CDCl_3$) δ 171.97, 155.67, 155.34, 86.01, 81.13, 78.74, 77.48, 77.16, 76.84, 74.55, 73.86, 70.17, 63.65, 63.60, 40.28, 34.80, 32.24, 29.59, 29.18, 28.86, 27.12.

1,8-octanedithiol Isomannide Polyurethane (C8 IMPU)

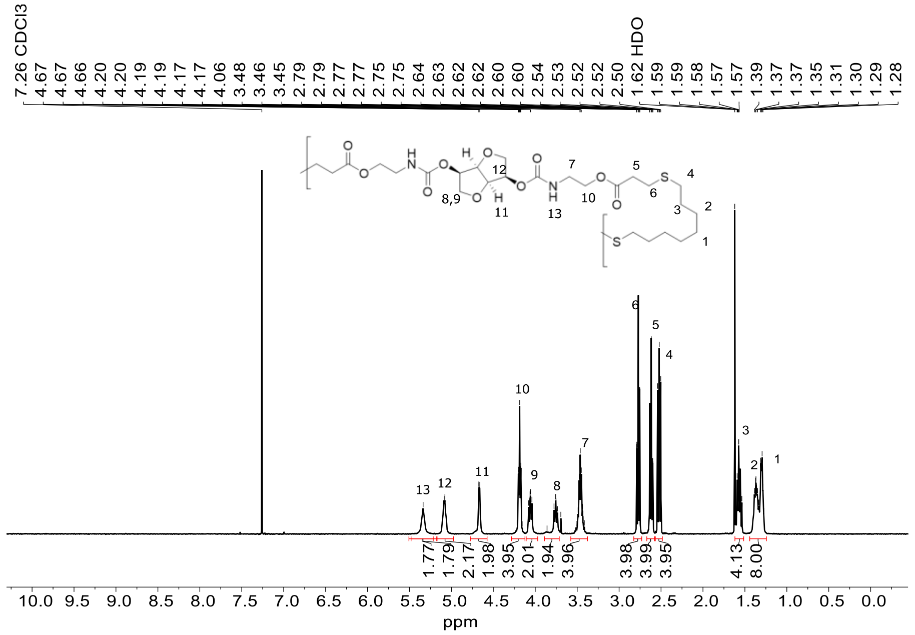

Figure S11. ^{1}H NMR spectra of 1,8-octanedithiol isomannide polyurethane (C8 IMPU) ^{1}H NMR (400 MHz, $CDCl_3$) δ 5.34 (s, 2H), 5.09 (s, 2H), 4.77 – 4.57 (m, 2H), 4.28 – 4.12 (m, 4H), 4.06 (dd, *J* = 9.1, 6.6 Hz, 2H), 3.89 – 3.71 (m, 2H), 3.46 (m, 4H), 2.77 (t, *J* = 7.2, 4H), 2.62 (t, *J* = 7.2, 4H), 2.57 – 2.48 (m, 4H), 1.62 – 1.51 (m, 4H), 1.44 – 1.24 (m, 8H).

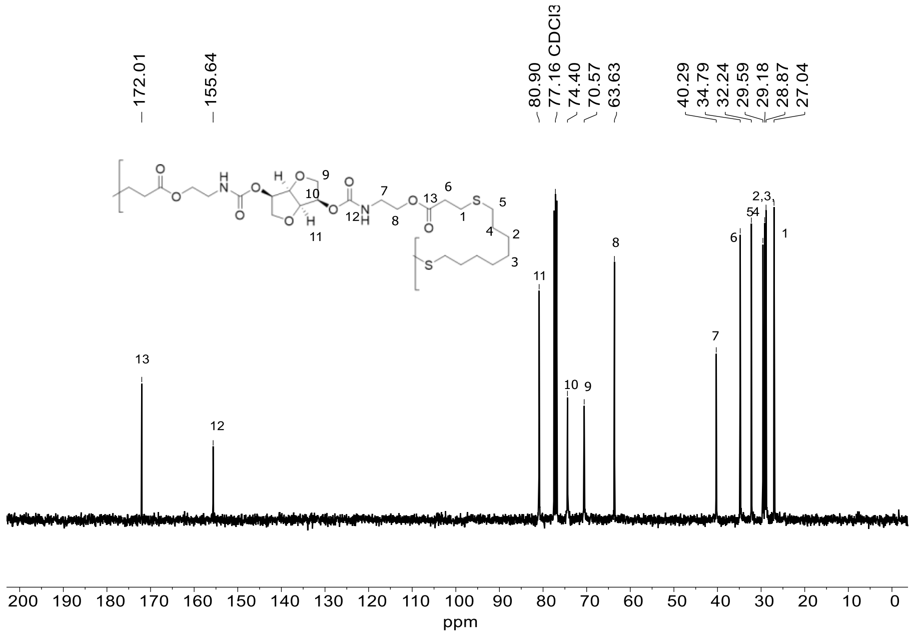

Figure S12. ^{13}C NMR spectra of 1,8-octanedithiol isomannide polyurethane (C8 IMPU) ^{13}C NMR (101 MHz, $CDCl_3$) δ 172.01, 155.64, 80.90, 77.16, 74.40, 70.57, 63.63, 40.29, 34.79, 32.24, 29.59, 29.18, 28.87, 27.04.

1,8-octanedithiol isosorbide polyester (C8 ISNU)

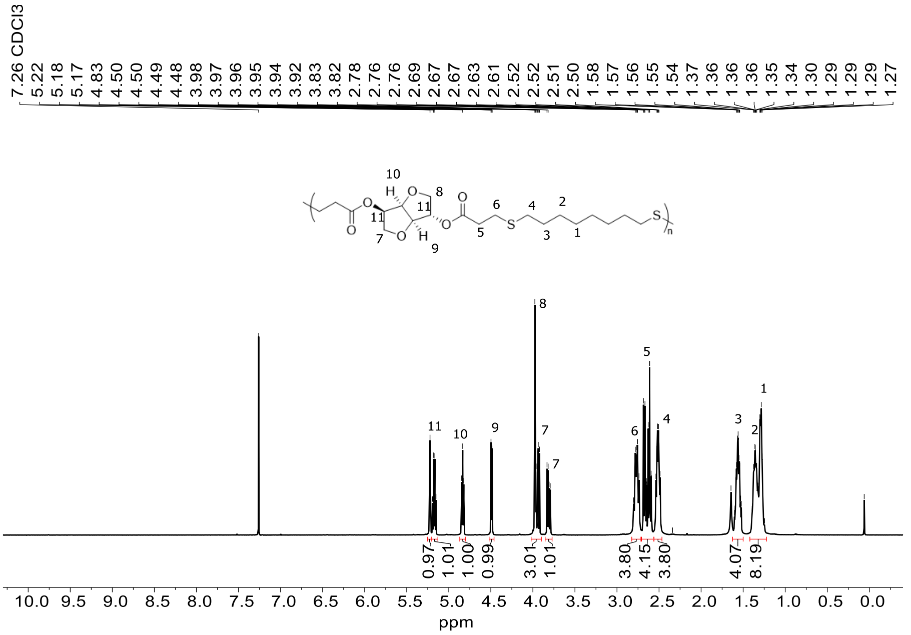

Figure S13. ^{1}H NMR spectra of 1,8-octanedithiol isosorbide polyester (C8 ISNU) (400 MHz, Chloroform-*d*) δ 5.24 – 5.21 (m, 1H), 5.20 – 5.14 (m, 1H), 4.87 – 4.81 (m, 1H), 4.49 (d, *J* = 4.8 Hz, 1H), 3.97 (d, *J* = 2.4 Hz, 2H), 3.94 (dd, *J* = 9.9, 5.9 Hz, 1H), 3.81 (dd, *J* = 9.9, 5.2 Hz, 1H), 2.84 – 2.71 (m, 4H), 2.69 – 2.59 (m, 4H), 2.57 – 2.46 (m, 4H), 1.61 – 1.52 (m, 4H), 1.40 – 1.32 (m, 4H), 1.31 – 1.24 (m, 4H).

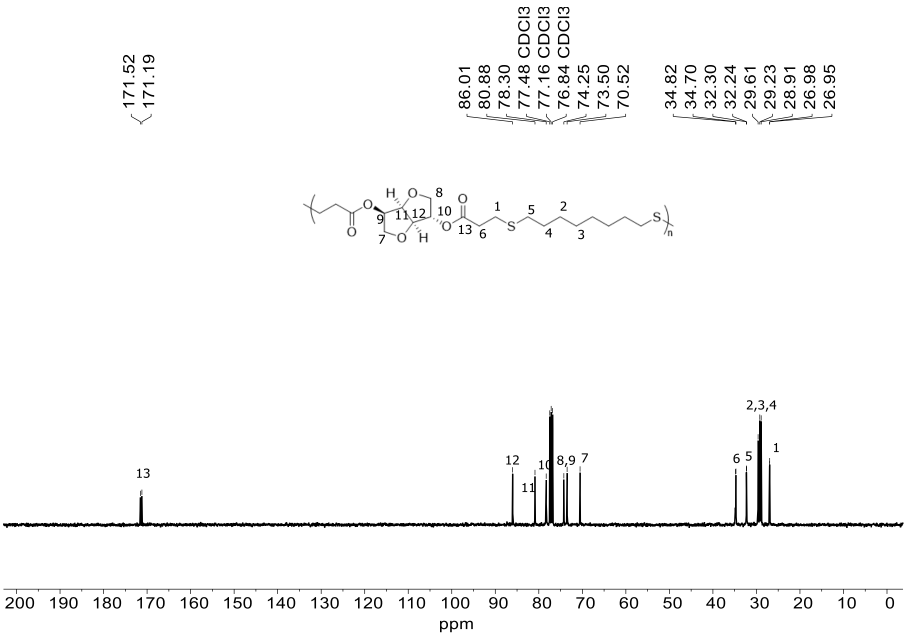

Figure S14. ^{13}C NMR spectra of 1,8-octanedithiol isosorbide polyester (C8 ISNU) (101 MHz, $CDCl_3$) δ 171.52, 171.19, 86.01, 80.88, 78.30, 74.25, 73.50, 70.52, 34.82, 34.70, 32.30, 32.24, 29.61, 29.23, 28.91, 26.98, 26.95.

1-octanedithiol-1,4-butanediol polyurethane (C8 SAT PU)

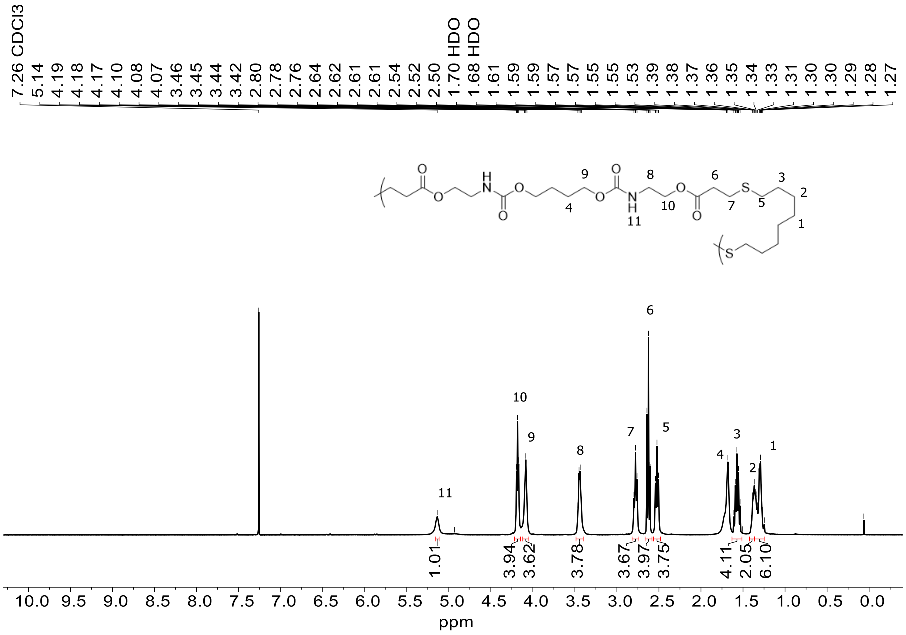

Figure S15. ^{1}H NMR spectra of 1-octanedithiol-1,4-butanediol polyurethane (C8 SAT PU) ^{1}H NMR (400 MHz, Chloroform-*d*) δ 5.14 (s, 1H), 4.18 (t, *J* = 5.3 Hz, 4H), 4.09 (d, *J* = 5.7 Hz, 4H), 3.44 (q, *J* = 5.4 Hz, 4H), 2.78 (t, *J* = 7.3 Hz, 4H), 2.62 (t, *J* = 7.0 Hz, 4H), 2.52 (m, 4H), 1.63 – 1.51 (m, 4H), 1.42 – 1.25 (m, 8H).

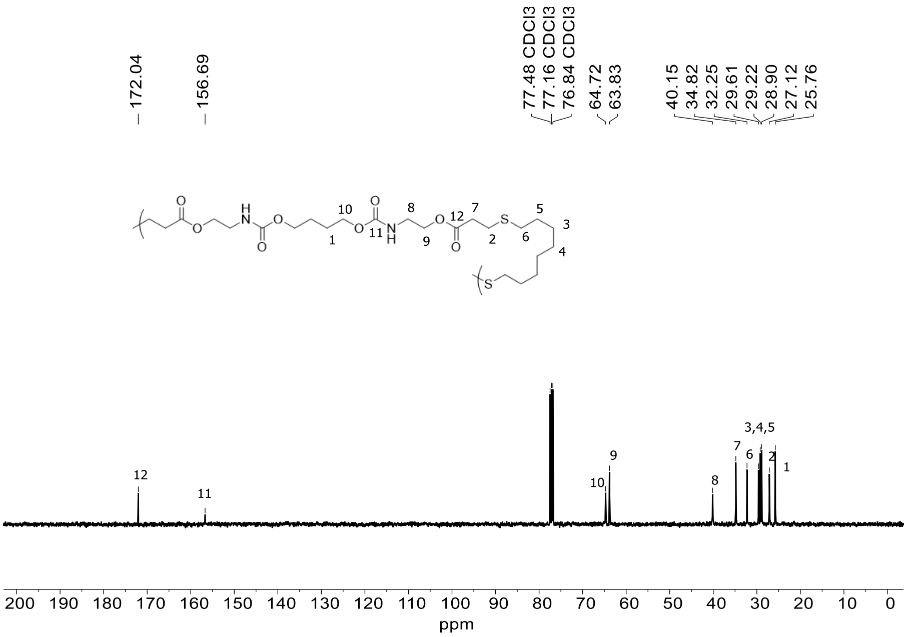

Figure S16. ^{13}C NMR spectra of 1-octanedithiol-1,4-butanediol polyurethane (C8 SAT PU) ^{13}C NMR (101 MHz, $CDCl_3$) δ 172.04, 156.69, 77.48, 77.16, 76.84, 64.72, 63.83, 40.15, 34.82, 32.25, 29.61, 29.22, 28.90, 27.12, 25.76.

SIZE-EXCLUSION CHROMATOGRAPHY

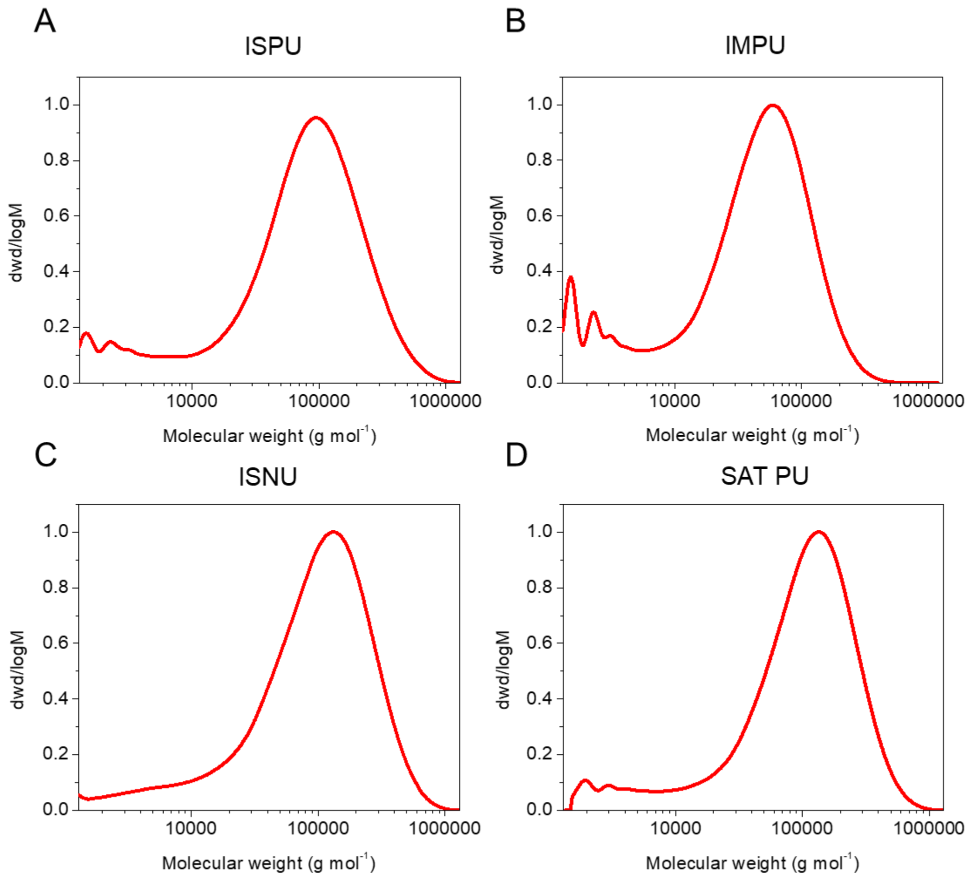

Figure S17. GPC chromatograms of (A) ISPU (B) IMPU (C) ISNU (D) SAT PU.

SUPPORTING INFORMATION

RHEOLOGY

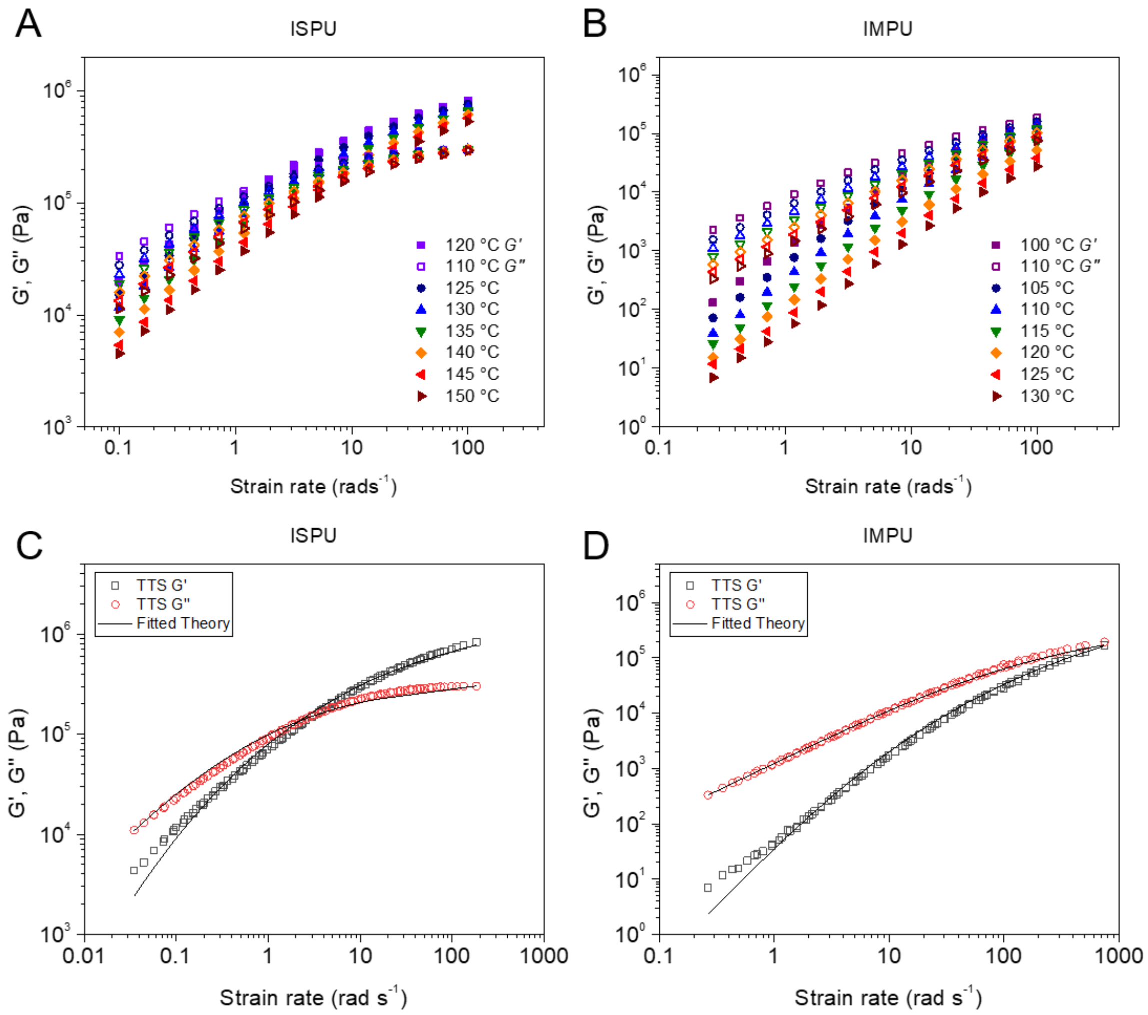

Figure S18. (A) Frequency sweeps of ISPU at discrete temperatures. (B) Frequency sweeps of IMPU at discrete temperatures. (C) Time-Temperature superposition of ISPU using WLF fitting reference temp is 130 °C. (D) Time-Temperature superposition of IMPU using WLF fitting reference temp is 130 °C. Data from the WLF overlay was fitted using double-reptation theory in the REPTATE software package.

Table S1. Entanglement molecular weight (M_w), plateau modulus (G_e), and rouse time (τ_e) of ISPU and IMPU.

Polymer	M_e (kDa)	G_e (GPa)	τ_e (μs)
ISPU	5.4	3.4	8.1
IMPU	5.7	5.3	3.9

DIFFERENTIAL SCANNING CALORIMETRY

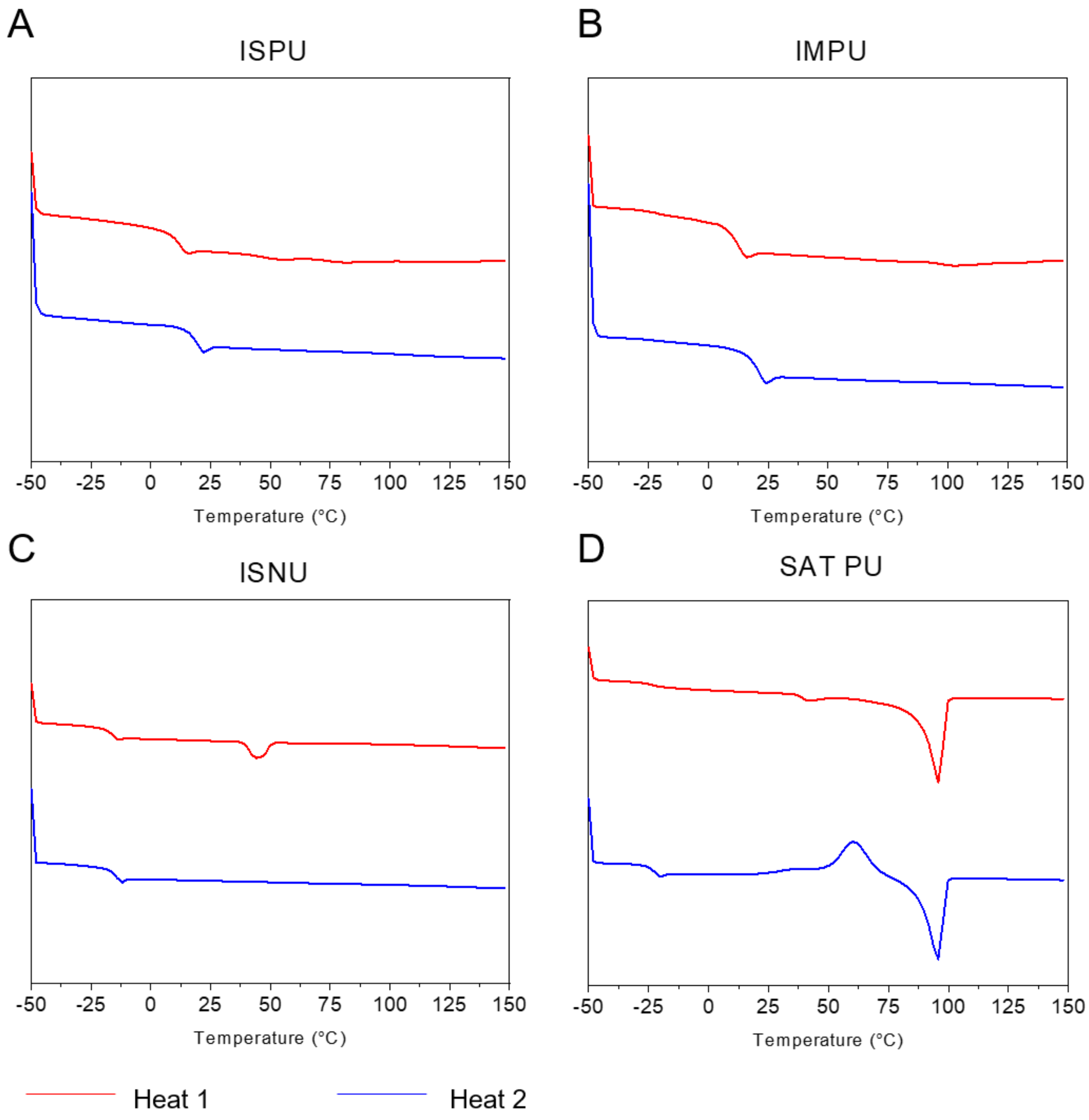

Figure S19. DSC thermograms of (A) ISPU (B) IMPU (C) ISNU and (D) SAT PU.

THERMOGRAVIMETRIC ANALYSIS

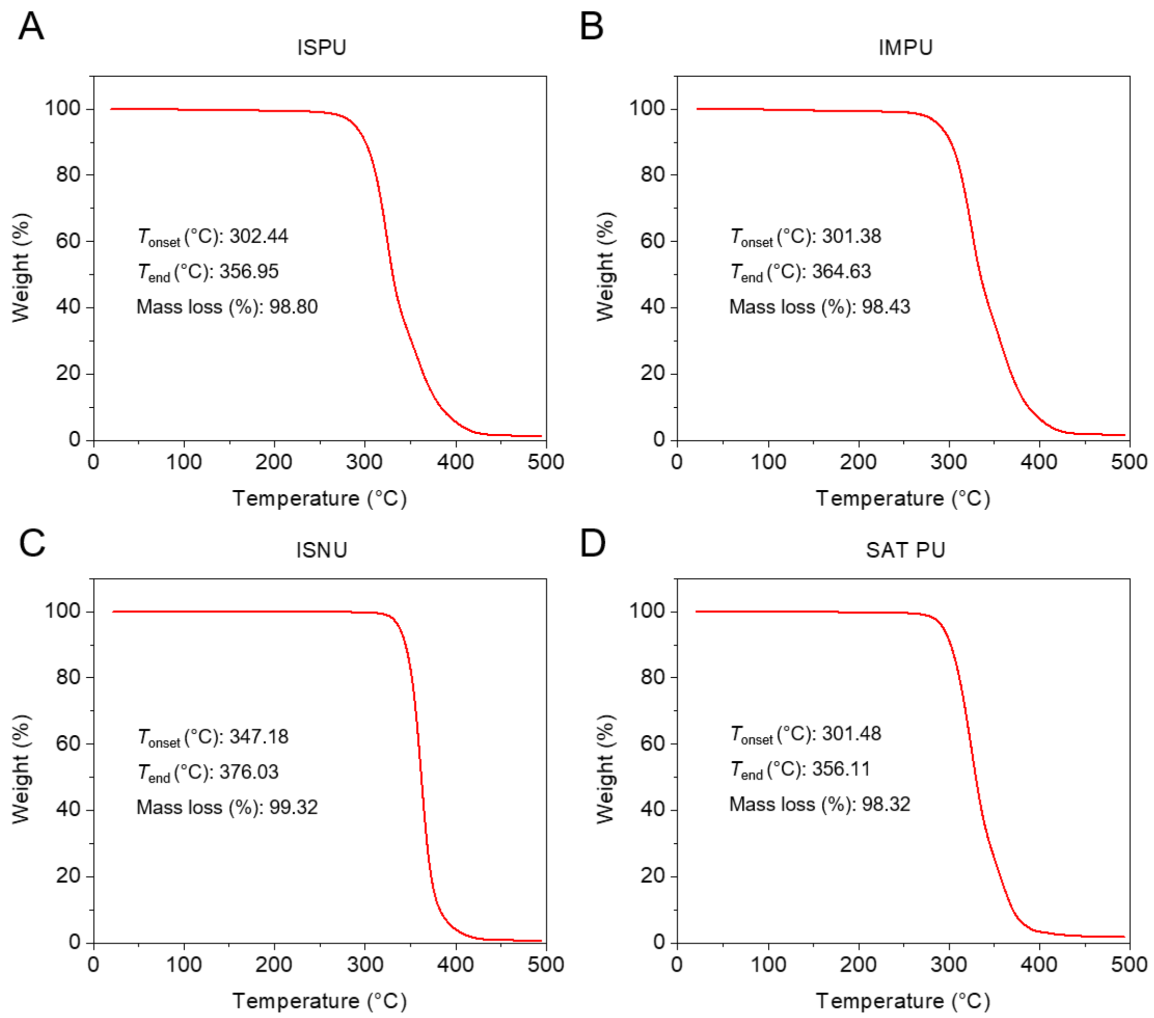

Figure S20. TGA thermograms of (A) ISPU (B) IMPU (C) ISNU and (D) SAT PU.

MECHANICAL CHARACTERIZATION

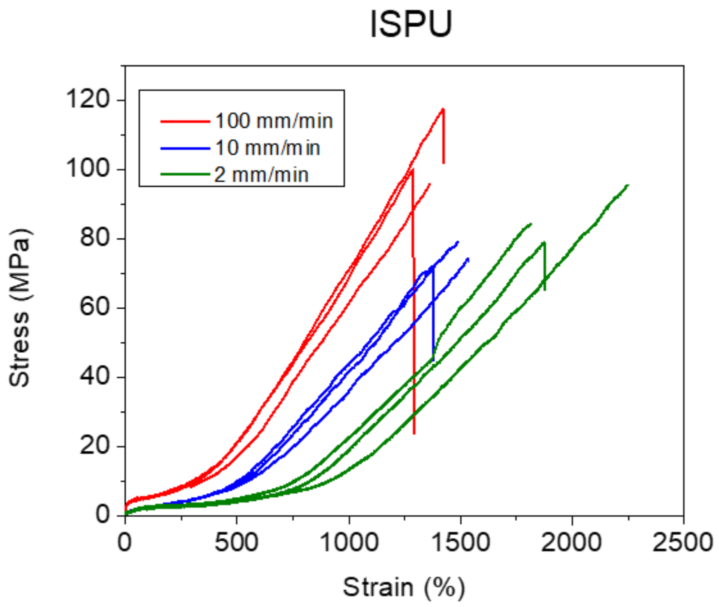

Figure S21. Stress vs. strain curves obtained by tensile testing of ISPU films, tested at rate of 2 mm min^{-1}, 10 mm min^{-1}, and 100 mm min^{-1} (22 °C, n = 3, annealed at 25 °C for 7 d after pressing).

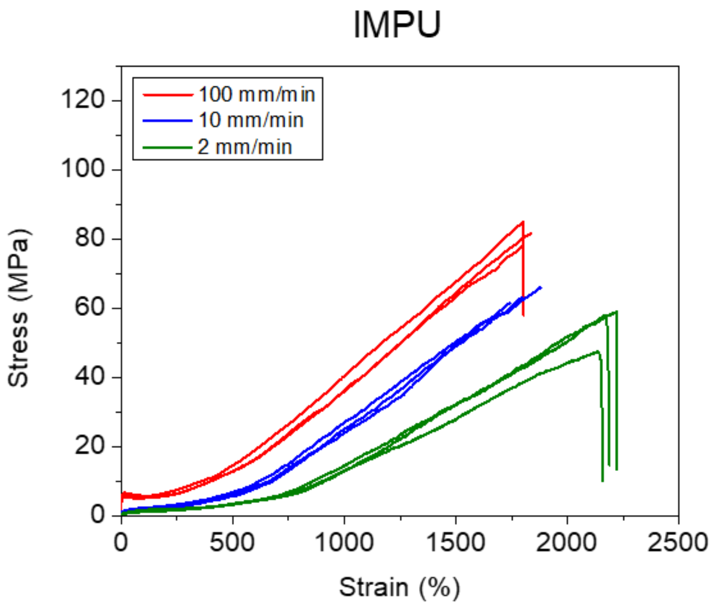

Figure S22. Stress vs. strain curves obtained by tensile testing of IMPU films, tested at rate of 2 mm min^{-1}, 10 mm min^{-1}, and 100 mm min^{-1} (22 °C, n = 3, annealed at 25 °C for 7 d after pressing).

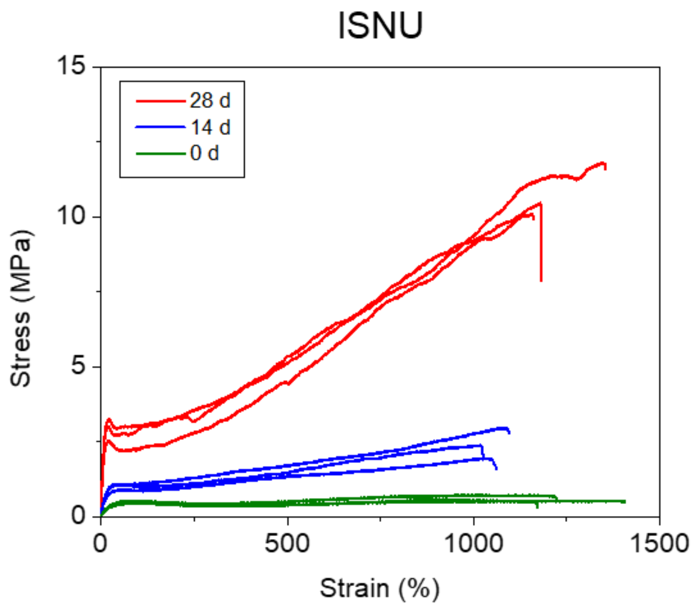

Figure S23. Stress vs. strain curves obtained by tensile testing of ISNU films, tested after annealing at 25 °C for 0 d, 14 d, and 28 d (22 °C, n = 3).

**Annealing time (days)**	**Stress at yield (MPa)**[a]	**Strain at Break (%)**[a]	**Stress at Break (MPa)**[a]	***E* (MPa)**[a]	***U*$_T$ (MJ/m^3)**[a]
0	-	1254 ± 123	0.58 ± 0.09	2.31 ± 0.35	6.14 ± 0.88
14	1.01 ± 0.1	1037 ± 33	2.44 ± 0.51	6.86 ± 1.33	16.8 ± 3.0
28	2.92 ± 0.38 ±	1226 ± 102	10.8 ± 0.9	46.3 ± 3.6	76.6 ± 14.74

Tables S2. Table of data summarizing tensile data of ISNU. [a] Values obtained from the analysis of tensile data. Standard deviation of samples taken as the uncertainty.

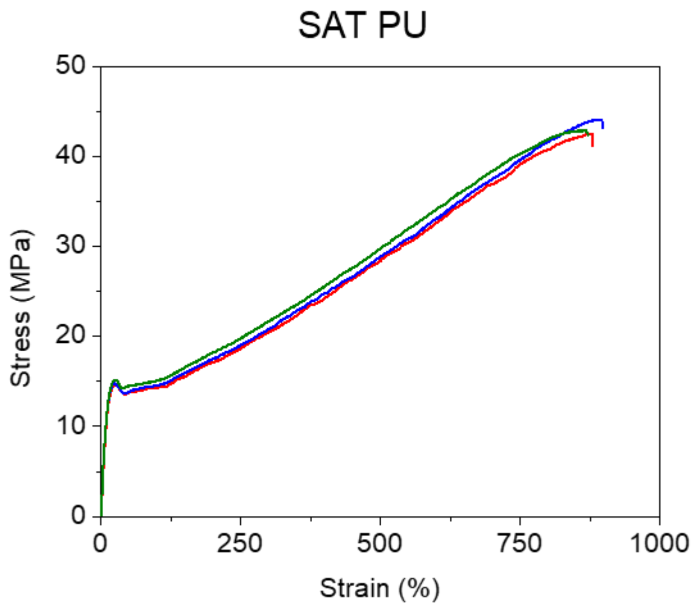

Figure S24. Stress vs. strain curves obtained by tensile testing of SAT PU films (22 °C, n = 3, annealed at 25 °C for 7 d after pressing).

OPTICAL TRANSPARENCY

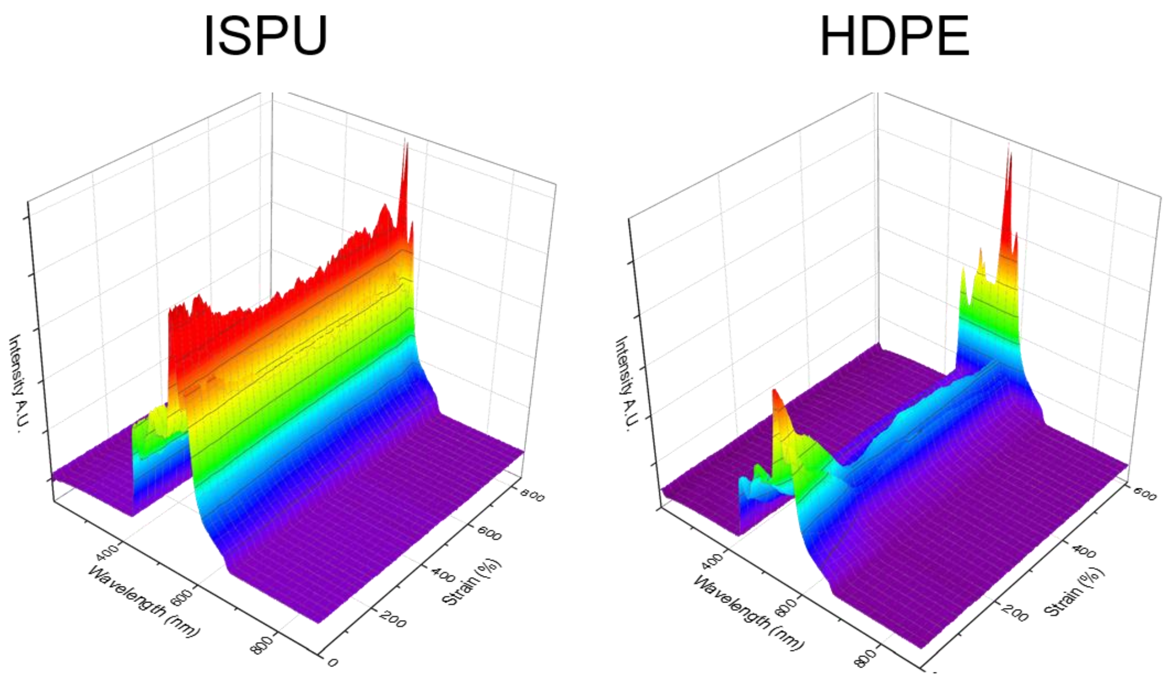

Figure S25. Absorbance of white light as it is passed through ISPU and HDPE as they are strained to 800% and 600% elongation respectively. HDPE undergoes strain-induced crystallization during deformation, resulting in decreased transmittance of light through the sample.

SAX and WAX

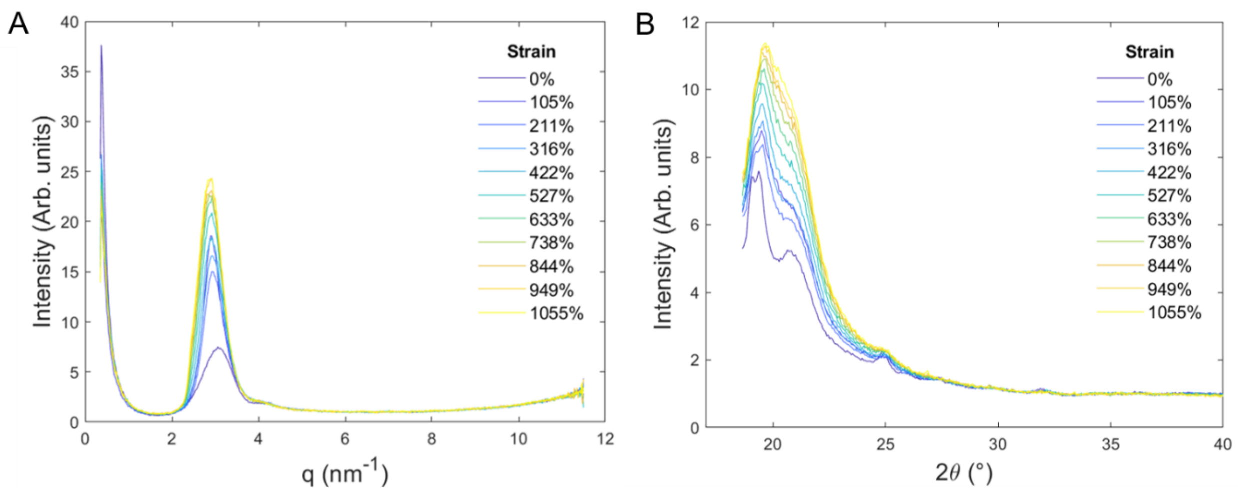

Figure S26. A) SAXS and B) WAXS of C8 ISNU measured under tensile elongation at 10 mm min^{-1} on dumbbell-shaped samples cut from thin films annealed for 28 days at 25 °C.

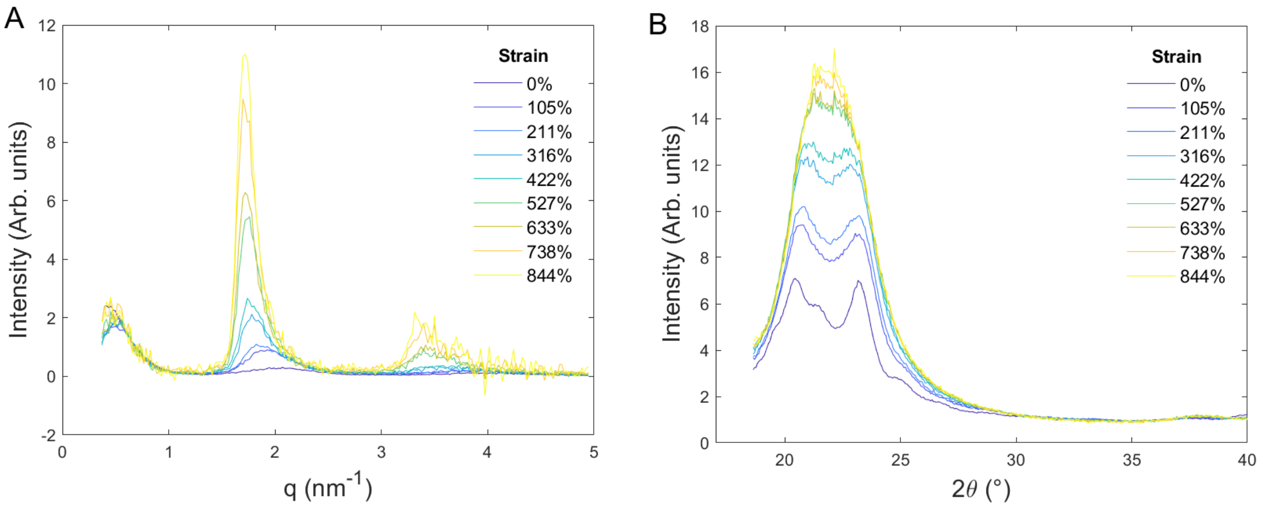

Figure S27. A) SAXS and B) WAXS of SAT PU measured under tensile elongation at 10 mm min^{-1} on dumbbell-shaped samples cut from thin films annealed for 28 days at 25 °C.

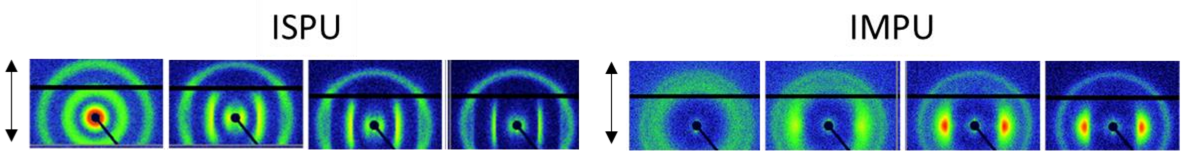

Figure S28. 2D SAXS patterns for ISPU (left) and IMPU (right) under tensile stress.

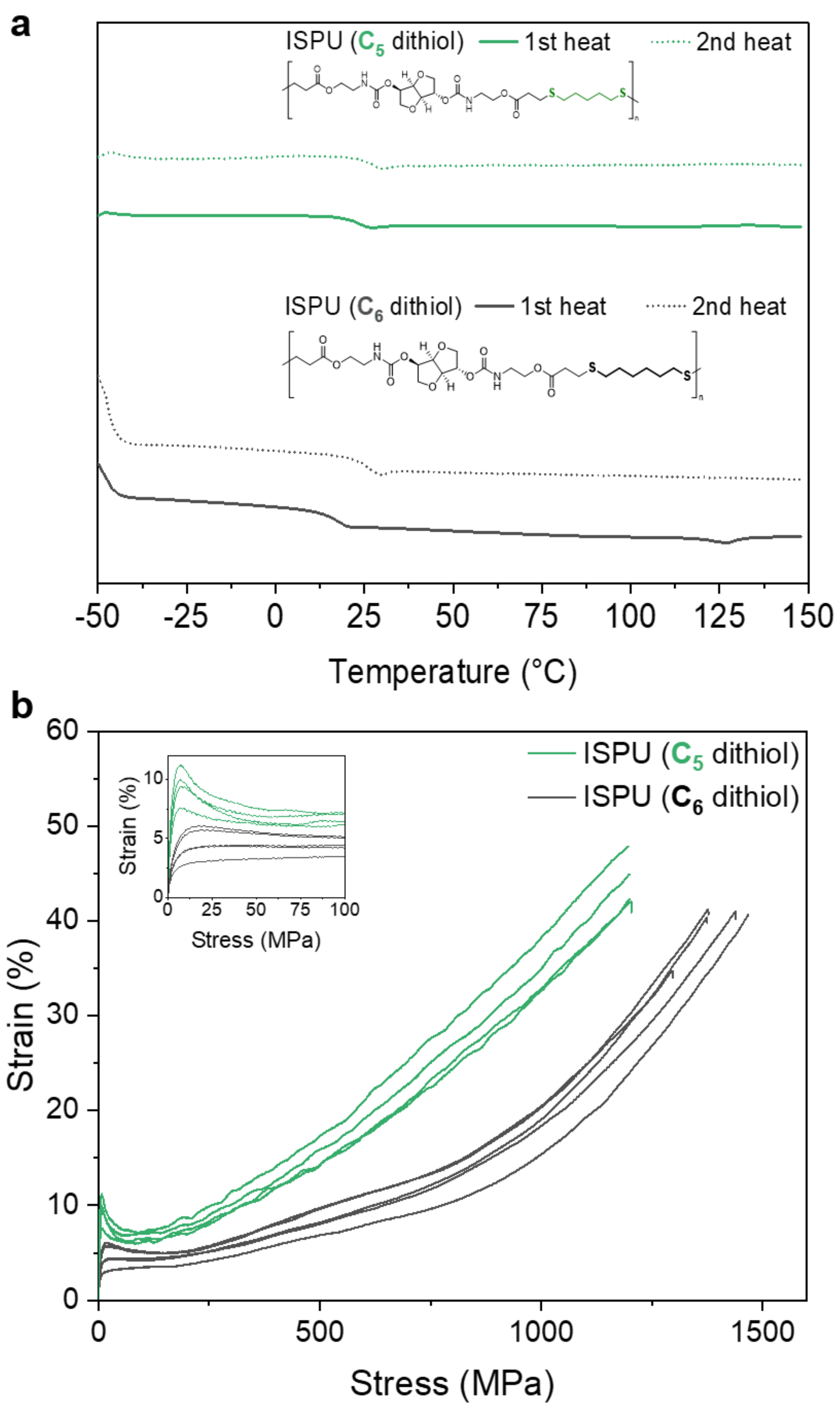

Figure S29. Thermomechanical analysis of ISPU with different dithiol comonomers (A) DSC thermograms and (B) Stress vs. strain curves obtained by tensile testing of films at 10 mm min^{-1} (22 °C, n > 3, annealed at 25 °C for 7 d after pressing).

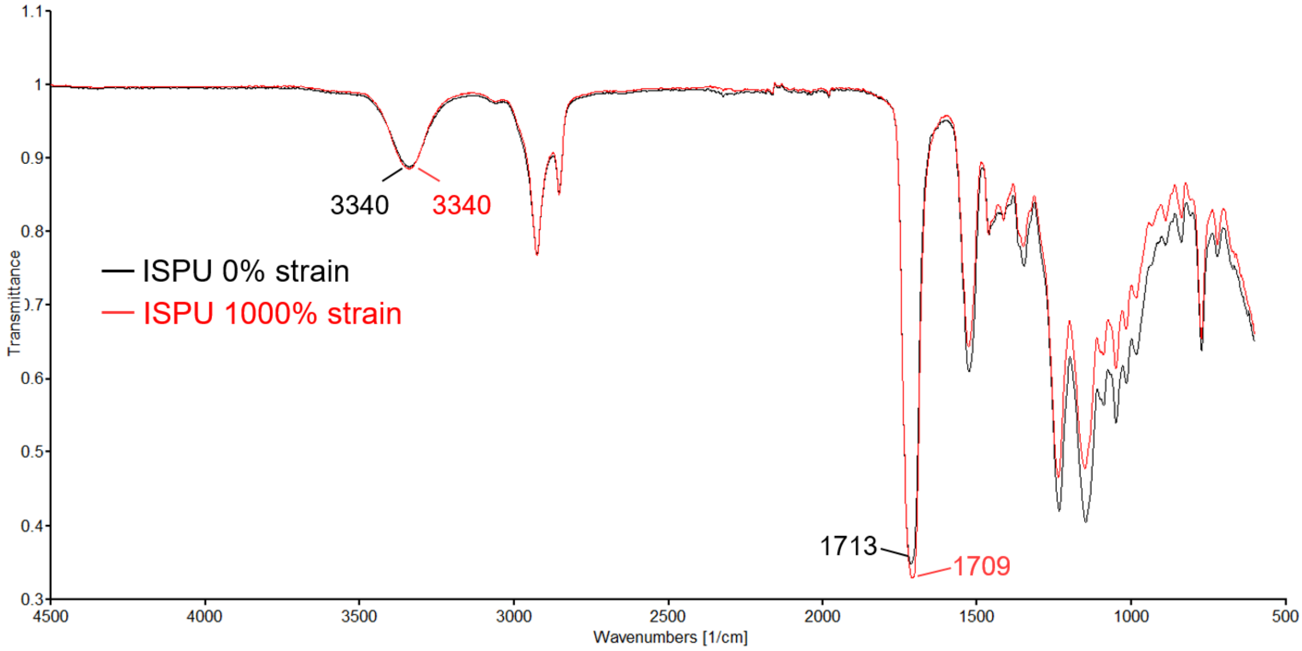

Figures S30. FTIR analysis of ISPU at 0% strain and 1000% strain.

SIMULATION DETAILS

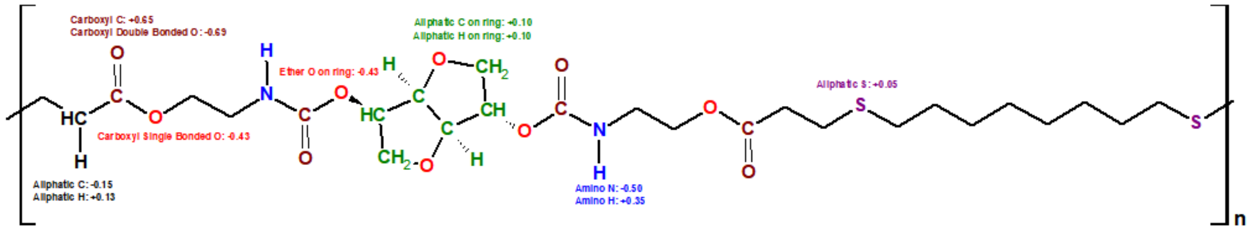

Figure S29. Partial charge distribution for the polymer repeat unit.

Table S3. Interaction Parameters

id	**Atom Types**	**Mass**(g/mol)	ϵ (kCal/mol)	σ(A)	**Description**
1	C	12.0107	0.086	3.39967	*sp*^{2} C in Carboxyl group
2	C3	12.0107	0.1094	3.39967	*sp*^{3} C in Aliphatic chain
3	HC	1.00794	0.0157	2.649533	H bonded to *sp*^{3} C
4	HN	1.00794	0.0157	1.069078	H bonded to N
5	N	14.0067	0.17	3.249999	*sp*^{2} N in amide groups
6	O	15.9994	0.21	2.959922	O double bonded in carboxyl group
7	OS	15.9994	0.17	3.000012	O in ester/ether bond
8	SS	32.065	0.25	3.563595	*sp*^{3} S in in thio-ester and thio-ether

id	**Bond Types**	k_r (kCal/mol A^{-2})	r_{eq} (A)	**Description**
1	C-C3	328.3	1.508	C-C bond between aliphatic C and carboxyl group
2	C3-N	478.2	1.345	N-C bond between aliphatic C to amino group N
3	C-O	648	1.214	O-C double bond in carboxyl group
4	C-OS	411.3	1.343	O-C single bond in carboxyl group
5	C3-C3	303.1	1.535	C-C bond between aliphatic C
6	C3-HC	337.3	1.092	C-H bond in aliphatic C chain
7	C3-N	330.6	1.46	C-N bond in amide group
8	C3-OS	301.5	1.439	C-O bond in ester bond
9	C3-S	225.8	1.821	C-S bond in thio-ether bond
10	HN-N	410.2	1.009	N-H bond in amide group

id	**Angle Types**	k_θ (kCal/mol rad^{-2})	θ_{eq} (degree)
1	C-C3-C3	63.8	110.53
2	C-C3-HC	47.2	109.68
3	C-N-C3	63.9	121.35
4	C-N-HN	49.2	118.46
5	C-OS-C3	63.6	115.14
6	C3-C-O	68	123.11
7	C3-C-OS	69.3	111.96
8	C3-C3-C3	63.2	110.63
9	C3-C3-HC	46.4	110.05
10	C3-C3-N	65.9	112.13
11	C3-C3-OS	67.8	108.42
12	C3-C3-S	61.1	112.69
13	C3-N-HN	46	116.78
14	C3-OS-C3	62.1	113.41
15	C3-S-C3	60.6	99.92
16	HC-C3-HC	39.4	108.35
17	HC-C3-N	49.8	109.5
18	HC-C3-OS	50.9	108.7
19	HC-C3-S	42.5	108.76
20	N-C-O	75.8	122.03
21	N-C-OS	74.7	115.25
22	O-C-OS	76.2	122.43

id	**Proper Dihedral Types**	k_d (kCal/mol)	*d*	*n*
1	C-C3-C3-HC	0.155556	1	3
2	C-C3-C3-S	0.155556	1	3
3	C3-C-OS-C3	2.7	-1	2
4	C3-C3-C3-C3	0.155556	1	3
5	C3-C3-C3-C3	0.18	1	3
6	C3-C3-C3-C3	0.25	-1	2
7	C3-C3-C3-C3	0.2	-1	1
8	C3-C3-C3-HC	0.155556	1	3
9	C3-C3-C3-HC	0.16	1	3
10	C3-C3-C3-OS	0.155556	1	3
11	C3-C3-C3-S	0.155556	1	3
12	C3-C3-N-C	0	1	2
13	C3-C3-N-C	0.5	-1	4
14	C3-C3-N-C	0.15	-1	3
15	C3-C3-N-C	0.53	1	1
16	C3-C3-N-HN	0	1	2
17	C3-C3-OS-C	0.383333	1	3
18	C3-C3-OS-C	0.383	1	3
19	C3-C3-OS-C	0.8	-1	1
20	C3-C3-OS-C3	0.383333	1	3
21	C3-C3-OS-C3	0.383	1	3
22	C3-C3-OS-C3	0.1	-1	2
23	C3-C3-S-C3	0.333333	1	3
24	HC-C3-C3-HC	0.155556	1	3
25	HC-C3-C3-HC	0.15	1	3
26	HC-C3-C3-N	0.155556	1	3
27	HC-C3-C3-OS	0.155556	1	3
28	HC-C3-C3-OS	0.25	1	1
29	HC-C3-C3-S	0.155556	1	3
30	HC-C3-N-C	0	1	2
31	HC-C3-N-HN	0	1	2
32	HC-C3-OS-C	0.383333	1	3
33	HC-C3-OS-C3	0.383333	1	3
34	HC-C3-S-C3	0.333333	1	3
35	N-C-OS-C3	2.7	-1	2
36	N-C3-C3-OS	0.155556	1	3
37	O-C-C3-C3	0	-1	2
38	O-C-C3-HC	0	-1	2

39	O-C-C3-HC	0.8	1	1
40	O-C-C3-HC	0.08	-1	3
41	O-C-N-C3	2.5	-1	2
42	O-C-N-HN	2.5	-1	2
43	O-C-N-HN	2.5	-1	2
44	O-C-N-HN	2	1	1
45	O-C-OS-C3	2.7	-1	2
46	O-C-OS-C3	2.7	-1	2
47	O-C-OS-C3	1.4	-1	1
48	OS-C-C3-C3	0	-1	2
49	OS-C-C3-HC	0	-1	2
50	OS-C-N-C3	2.5	-1	2
51	OS-C-N-HN	2.5	-1	2
52	OS-C3-C3-OS	0.155556	1	3
53	OS-C3-C3-OS	0.144	1	3
54	OS-C3-C3-OS	1.175	1	2

id	**Improper Dihedrals**	k_d (kCal/mol)	d	n	**Description**
1	C3-O-C-OS	10.5	-1	2	CCOO plane
2	N-O-C-OS	10.5	-1	2	NCOO plane

REFERENCES

1. Wang, J.; Wolf, R. M.; Caldwell, J. W.; Kollman, P. A.; Case, D. A. Development and testing of a general amber force field, *J. Comput. Chem.* **2004,** 25, (9), 1157-1174.
2. Humphrey, W.; Dalke, A.; Schulten, K. VMD: Visual molecular dynamics, *J. Mol. Graph. Model.* **1996,** 14, (1), 33-38.
3. Brown, W. M.; Kohlmeyer, A.; Plimpton, S. J.; Tharrington, A. N. Implementing molecular dynamics on hybrid high performance computers – Particle–particle particle-mesh, *Comput. Phys. Commun.* **2012,** 183, (3), 449-459.
4. Hockney, R. W.; Eastwood, J. W., *Computer simulation using particles*. Taylor & Francis, Inc.: 1988; p 564.
5. Plimpton, S. Fast Parallel Algorithms for Short-Range Molecular Dynamics, *J. Comput. Phys.* **1995,** 117, (1), 1-19.
